# A comparison of RNA-Seq data preprocessing pipelines for transcriptomic predictions across independent studies

**DOI:** 10.1186/s12859-024-05801-x

**Published:** 2024-05-08

**Authors:** Richard Van, Daniel Alvarez, Travis Mize, Sravani Gannavarapu, Lohitha Chintham Reddy, Fatma Nasoz, Mira V. Han

**Affiliations:** 1 School of Life Sciences, University of Nevada Las Vegas, Las Vegas, NV USA; 2 Department of Computer Science, University of Nevada Las Vegas, Las Vegas, NV USA; 3 Nevada Institute of Personalized Medicine, Las Vegas, NV USA; 4 Icahn School of Medicine at Mount Sinai, Institute for Genomic Health, New York City, NY USA

**Keywords:** RNA-Seq, Classification, Cancer, Batch effect correction, Normalization, Data scaling

## Abstract

**Background:**

RNA sequencing combined with machine learning techniques has provided a modern approach to the molecular classification of cancer. Class predictors, reflecting the disease class, can be constructed for known tissue types using the gene expression measurements extracted from cancer patients. One challenge of current cancer predictors is that they often have suboptimal performance estimates when integrating molecular datasets generated from different labs. Often, the quality of the data is variable, procured differently, and contains unwanted noise hampering the ability of a predictive model to extract useful information. Data preprocessing methods can be applied in attempts to reduce these systematic variations and harmonize the datasets before they are used to build a machine learning model for resolving tissue of origins.

**Results:**

We aimed to investigate the impact of data preprocessing steps—focusing on normalization, batch effect correction, and data scaling—through trial and comparison. Our goal was to improve the cross-study predictions of tissue of origin for common cancers on large-scale RNA-Seq datasets derived from thousands of patients and over a dozen tumor types. The results showed that the choice of data preprocessing operations affected the performance of the associated classifier models constructed for tissue of origin predictions in cancer.

**Conclusion:**

By using TCGA as a training set and applying data preprocessing methods, we demonstrated that batch effect correction improved performance measured by weighted F1-score in resolving tissue of origin against an independent GTEx test dataset. On the other hand, the use of data preprocessing operations worsened classification performance when the independent test dataset was aggregated from separate studies in ICGC and GEO. Therefore, based on our findings with these publicly available large-scale RNA-Seq datasets, the application of data preprocessing techniques to a machine learning pipeline is not always appropriate.

**Supplementary Information:**

The online version contains supplementary material available at 10.1186/s12859-024-05801-x.

## Background

Classical cancerous tissue classification was primarily based on the clinical interpretation of the morphological characteristics of a tumor specimen, however technological advancements made possible molecular approaches to prediction of tissue of origin for cancer that utilize genome-wide expression data [1]. Machine learning applications can be used to relate a patient's gene expression measurements to an endpoint variable of interest such as the patient's cancer type [2]. There is a wealth of publicly available human expression data for thousands of cancer patients representing over 30 cancer types because of the efforts by the TCGA consortium [3]. Other large efforts by various consortia such as GTEx, ICGC, and GEO have also deposited transcriptomics datasets into freely accessible databases derived from diverse tissues and conditions [4–6]. These open repositories provide RNA-Seq data sources that can be integrated with machine learning strategies to construct a classifier model for the prediction of cancer tissue types. Due to the large size and complexity of the RNA-Seq datasets, machine learning is a practical means to make sense of this abundance of data. To extract useful biological information from gene expression data, we employed the support vector machine (SVM), a popular machine learning model among other researchers working with TCGA data [7].

Data preprocessing tasks such as normalization, batch effect removal, and data scaling are crucial for classifier inputs and will affect predictive performance estimates. Normalization is an essential step in RNA-Seq analysis that adjusts global properties of raw expression measurements to minimize systematic variations; its purpose is to allow the expression levels to be appropriately compared across samples with differences only due to biological factors [8]. Batch effects are the unwanted variation between groups (i.e., batches) of samples and unrelated to the outcome of interest [9]. The impact of batch effects is particularly severe in studies that measure the expression levels of thousands of genes at once, such as RNA-Seq. This could be due to the numerous sources of variation in the multi-step process of data generation for the high-throughput technology [10]. Data scaling is another data preprocessing step often necessary to put the feature set into a common frame, before the rescaled dataset is used as input in a classifier. Features in a similar range allow each feature to contribute equally to the impact on the model performance [11].

Despite the wealth of RNA-Seq experiments accumulated, RNA-Seq data are largely utilized for unsupervised learning purposes in identifying subtypes or clusters, and not utilized enough for classification. The main reason for this limited utility of RNA-seq datasets for classification or prediction, especially in the clinical setting, is the prevalence of batch effects in these datasets. The variation originates from various sources in the multi-step process of generating the RNA-seq data, including variables related to the sample conditions, sample collection including ischemic time, RNA enrichment protocol, RNA quality, cDNA library preparation, sequencing platform, sequencing quality, and total sequencing depth [12]. Despite the potential for extensive unwanted variation, the measurement at the final stage is digital and high-resolution, ensuring that all the latent variation is reliably captured and reflected in the data. This becomes a serious issue for classification, leading to inflated performance measures in case of shared batch effects between training and test datasets. It also results in low generalization against unseen test data in case of unique batch effects and distributional differences in the newly generated data [13].

There have been previous approaches that try to correct for specific sources of variation. For example, several studies have devised models to correct for the sequence specific biases that arise from the preparation of cDNA libraries [14, 15]. Another study has proposed a uniform alignment and quantification pipeline to address the variation arising from the computational analysis [16]. Alternatively, there have been efforts to correct for unwanted variation in a general and agnostic approach through batch correction. It is generally advised to apply batch effect adjustment in cross-batch prediction, as the resulting performance is better than no batch effect removal at all. This has been demonstrated by evaluating many types of batch effect correction algorithms on microarray datasets [17]. There is no clear consensus on the most reliable batch effect correction algorithm, as no single method has consistently outperformed others across all performance metrics [18]. Quantile normalization has previously been used for microarray data by assimilating the test data to the training data before applying prediction rules to improve cross-study performance [19]. Technical phenotype information on expression data, when available, can be incorporated into analyses to improve performance [20]. Alternative models of ComBat, a popular batch effect correction algorithm, have been developed. The original ComBat model pools all samples to estimate the mean and variance batch effect resulting in transformations of both the training and test datasets [21]. The reference-batch ComBat method uses one batch as a reference for the batch effect adjustment of the non-reference batch [22]. In this context, the training set (reference batch) is fixed and the test dataset (non-reference batch) will be corrected toward the distribution of the unadjusted training data. This approach, in theory, should improve the performance of the future prediction of unseen test samples with new batch effects.

Here we evaluate various preprocessing methods for cross-study classification performance using a case study of tissue prediction trained on a TCGA and tested against GTEx or ICGC/GEO datasets. To the best of our knowledge, there is currently no comparison study that empirically assesses the effectiveness of data preprocessing techniques outlined in this paper on the predictive performance of the molecular classification of cancerous tissue across large-scale RNA-Seq datasets. We hypothesize that certain combinations of these data preprocessing techniques applied to a high throughput dataset will be able to reduce prediction error as compared to a baseline model. Our specific aims for this work are to quantify the differences in performance between utilizing unmodified transcriptomic data versus the preprocessed datasets and understand which data preprocessing procedures are essential to this effort.

**Fig. 1:**
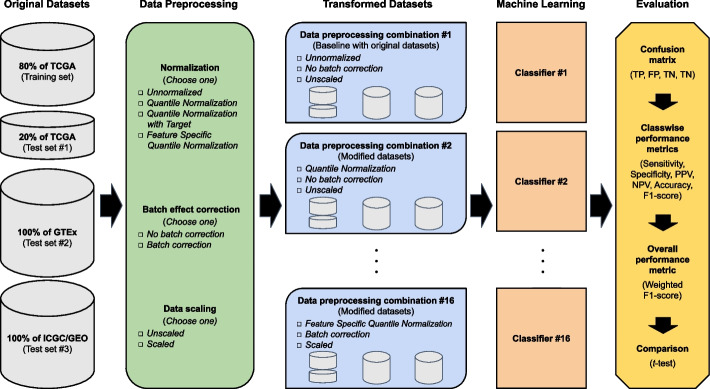
Flow chart of data preprocessing, machine learning, and evaluation approaches. Large-scale RNA-Seq datasets were freely available to be obtained from TCGA, GTEx, ICGC, and GEO and assigned to training and test sets. The original datasets are part of data preprocessing combination #1 and serve as the 'baseline' for comparison. The transformed datasets of data preprocessing combinations #2 through #16 are based on various combinations of normalization (*Unnormalized*, *Quantile Normalization*, *Quantile Normalization with Target*, and *Feature Specific Quantile Normalization*), batch effect correction (*No batch effect correction or Batch effect correction*), and data scaling (*Unscaled* or *Scaled*) procedures applied to the original datasets. Each of the data preprocessing combinations is used to build an associated machine learning classifier

To this end, we set out to construct and evaluate machine learning pipelines based on various combinations of normalization, batch effect correction, and data scaling applied to the original datasets (Fig. 1).

Understanding the effects of the data preprocessing procedures on our pipelines for tissue of origin classification is necessary to obtain better performance estimates. This can provide valuable insight to other researchers building tools for multiclass predictors using high-throughput molecular data for predicting tissue of origins.

## Materials and methods

All data analyses were performed using Bash, R, and Python in Jupyter Notebooks [23].

### Genome-wide expression datasets

We downloaded publicly available RNA-Seq data files representing a panel of 14 malignancies from the Genomic Data Commons for TCGA human expression data, composed of 7192 primary tumor and 678 normal tissue samples for a total of 7870 samples; and V7 release of GTEx that contained 3340 matching healthy tissue samples [3, 4]. Only TCGA cancer types with more than 100 samples were considered sufficient for the learning phase of the training set to use in the machine learning classifier (*see below*). Note that we use the labels of TCGA to denote the tissue of origin, although the tissues can be both cancerous or non-cancerous depending on the study. The kidney cancers of TCGA were coalesced into one tissue type (i.e., KICH, KIRC, KIRP → KIRC). Colon and rectum cancers were merged as well (i.e., COAD, READ → COAD). ESCA and STAD samples were also integrated into a single tissue type (i.e., ESCA, STAD → GI) based on the similarity of their molecular profiles in these adjacent organs [24]. GI cancer samples were filtered to exclude esophageal squamous cell carcinoma, Epstein-Barr virus, and undifferentiated cancer types [25]. PAAD cancers were filtered to only include the samples that were curated to be true pancreatic ductal adenocarcinoma [26]. Additionally, we obtained ICGC and GEO RNA-Seq data files for 674 cancer and 202 non-cancer samples representing six tissue types, then combined the two datasets (hereafter referred to as "ICGC/GEO") for a total of 876 samples [5, 6]. A summary of the GEO datasets and the alignment tool used for each study is provided in Table S1. Reads for the TCGA, GTEx, and ICGC consortia were mapped to the human reference genome with STAR [27]; ICGC also used HISAT2 for alignment [28].

### Training and testing datasets

We performed a commonly used 80:20 split of the TCGA dataset: 80% of the TCGA data (6295 samples) were randomly assigned for training, while the remaining 20% (1575 samples) were used for internal evaluation testing [29]. Samples were drawn proportionally for each tissue type without replacement. Additionally, we tested against two independent test sets: one consisting of 100% of the GTEx dataset (3340 samples) and the other made up of 100% of the ICGC/GEO dataset (876 samples). A summary of the number of RNA-Seq samples for each tissue in the training and testing sets is provided in Table 1.

**Table 1:** Summary of cancer tissue types and number of RNA-Seq samples used in this study

Abbreviation	TCGA name	Tissue type	Training set	Test set #1	Test set #2	Test set #3
			(80% of TCGA)	(20% of TCGA)	(100% of GTEx)	(100% of ICGC/GEO)
			Number of cancer/normal samples	Number of cancer/normal samples	Number of normal samples	Number of cancer/normal samples
BLCA	Bladder urothelial carcinoma	Bladder	339	85	11	
BRCA	Breast invasive carcinoma	Breast	937	234	304	
CESC	Cervical squamous cell carcinoma and endocervical adenocarcinoma	Cervix	239	60	6	
COAD	Colon adenocarcinoma	Colon	533	133	281	
GI	Esophageal carcinoma and Stomach adenocarcinoma	Gastrointestinal (Esophagus/Stomach)	370	93	706	15
HNSC	Head and neck squamous cell carcinoma	Salivary glands	430	108	101	
KIRC	Kidney renal clear cell carcinoma	Kidney	805	201	48	77
LIHC	Liver hepatocellular carcinoma	Liver	334	84	187	349
LUAD	Lung adenocarcinoma	Lung	737	184	470	85
PAAD	Pancreatic adenocarcinoma	Pancreas	122	31	263	342
PCPG	Pheochromocytoma and paraganglioma	Adrenal gland	123	31	204	
PRAD	Prostate adenocarcinoma	Prostate	426	106	158	8
THCA	Thyroid carcinoma	Thyroid gland	442	111	486	
UCEC	Uterine corpus endometrial carcinoma	Uterus	458	114	115	
		(TOTAL)	6295	1575	3340	876

### Gene expression units and number of genes used for features

We obtained gene expression values in units of transcripts per million and then transformed the expression values to the logarithm base 2 scale [30, 31]. We found 50,370 genes belonging to TCGA, GTEx, and ICGC/GEO datasets by finding the common ENSEMBL gene IDs. We determined 253 genes with zero expression across all samples in the training set and filtered out these genes from all training and test sets to ultimately use 50,117 expressed genes as features for downstream analyses.

### Data preprocessing combinations of RNA-Seq datasets

We investigated a total of 16 data preprocessing combinations based on variations of normalization (*Unnormalized*, *Quantile Normalization [QN]*, *Quantile Normalization with Target [QN-Target]*, or *Feature Specific Quantile Normalization [FSQN]*), batch effect correction (*No batch correction or Batch correction*), and data scaling (*Unscaled* or *Scaled*). The RNA-Seq datasets, either the original or modified, include the training set (80% of TCGA) and test sets (20% of TCGA, 100% of GTEx, and 100% of ICGC/GEO). Data preprocessing combination #1 (*Unnormalized, No batch correction, and Unscaled)* is made up of the original RNA-Seq datasets and serves as the baseline for comparison to each set of modified RNA-Seq datasets from data preprocessing combinations #2 through #16.

### Normalization procedures

We considered three types of normalization steps to be applied on the original datasets. First, quantile normalization (QN), where each training and testing dataset was separately transformed to have identical distribution by replacing the expression level value with the ranked means according to their ranks within each sample. Second, quantile normalization with target (QN-Target), required that we transform the training set (i.e., the target) with QN and then the test datasets were transformed to be identical in distribution to the target distribution. For QN and QN-Target, we utilized the normalize.quantiles and normalize.quantiles.use.target functions, respectively, from the R package preprocessedCore [32]. Third, for feature specific quantile normalization (FSQN), we used the quantileNormalizeByFeature function of the R package FSQN to transform each feature of the test sets based on its corresponding feature in the training set [33].

### Batch effect correction procedures

Data preprocessing combinations that included batch effect correction were performed after normalization. Of the TCGA expression data, a type of batch effect we termed "protocol batch effect" arose from the use of different processing centers. Samples were processed at either British Columbia Cancer Michael Smith Genome Sciences Centre (BCGSC), which only included GI samples, or University of North Carolina (UNC) for samples of every other tissue type. We obtained the batch information of the sequencing center for the TCGA samples by using the R package TCGAutils to convert universal unique identifiers to TCGA barcodes [34]. Some notable differences in protocol included mRNA isolation, number of reads generated, number of reads discarded, and read length. For BCGSC, the MultiMACS mRNA isolation kit was used to generate a median of 227 million reads, of which 177 million were discarded, with 75 bps per read. For UNC, the TruSeq RNA Library Prep kit was used to generate a median of 149 million reads, of which 97 million were discarded, with 48 bps per read. Other types of batch effect are attributed to the data on disease state of cancer versus non-cancer samples (“disease batch effect”) and inter-project TCGA versus GTEx samples (“consortium batch effect”). The different batch effect types present in the current study are depicted in Figure S1. A separate approach was used to adjust for each type of batch effect we defined, executed in succession (Figure S2).

### Batch effect correction algorithms

We investigated three batch effect correction algorithms applied on the RNA-Seq datasets with the fixed constraints of *Unnormalized* and *Unscaled* data preprocessing procedures. First, we called the removeBatchEffect function from the R package limma to modify both the training and testing datasets by removing batch effects from the gene expression data [35]. Second, we used the ComBat function from the SVA package in R, with default parameters, to transform the training and testing datasets due to known batches [21]. Third, we utilized a variation of the ComBat function known as "reference-batch ComBat" to indicate a reference dataset that is most representative [22]. In this approach the specified reference batch (training dataset) remained unchanged. Conversely, only the gene expression values for the non-reference batch (testing dataset) were corrected toward the distribution of the fixed reference batch. We set the ref.batch option to the batches containing the highest number of samples for each of the three types of batch effect (*UNC, cancer*, and TCGA) as the reference datasets to correct for the protocol, disease, and consortium batch effect types, respectively.

### Batch effect adjustment approach for different batch types

Once we determined that limma was the best performing batch correction algorithm when GTEx served as the independent test set (*see Results*), we further investigated the specific types of batch effect that were necessary to be removed to achieve an increase in overall performance. With the fixed constraints of limma as the batch correction algorithm, and *Unnormalized* and *Unscaled* data preprocessing procedures, we explored all possible variations. This included adjustments for each of the batch types separately (protocol batch effect only, disease batch effect only, consortium batch effect only) and in combinations (protocol and disease batch effects; protocol and consortium batch effects; disease and consortium batch effects; protocol, disease, and consortium batch effects).

### Data scaling procedures

Data preprocessing combinations subject to data scaling took place after normalization and batch effect correction steps. We utilized the MinMaxScaler class of scikit-learn Python package with the feature range of 0–1 [36]. The data scaling factors determined for the training set were also applied on the test set. Separately determining scaling factors for the training and test datasets would generate an erroneous test set for downstream analysis [37].

### Machine learning algorithm

For our machine learning algorithm, we used SVM that aim to find a hyperplane capable of separating the data with the widest possible margin [38]. We utilized the SVC class of scikit-learn with the class weight parameter set to "balanced" to adjust weights inversely proportional to class frequencies [39].

### Dimension reduction techniques

We performed principal component analysis (PCA), a linear feature reduction technique, on the training dataset to obtain a lower-dimensional feature set, and then built a classifier with this new set of features [40]. Before applying the fitted model, the test dataset was transformed to the same PCA feature space of the training data. We also constructed visualizations based on the top two principal components that explain the most variance in the gene expression datasets. Furthermore, we applied t-distributed stochastic neighbor embedding (t-SNE) and uniform manifold approximation and projection (UMAP), non-linear dimension reduction techniques, to construct complementary low-dimensional representations of the original high-dimensional datasets [41, 42].

### Cross-validation protocol

We performed nested cross-validation for hyperparameter tuning using a grid search with *k*-fold cross-validation [43]. To prevent information leakage from the testing set into the training set, we applied dimension reduction techniques after cross-validation on the training sets of each fold rather than before which could have led to overly optimistic performance metrics [13]. The grid search optimization algorithm uses multiple trial-and-error processes of the hyperparameters settings, exploring the following possible configurations to achieve optimal performance estimates [44]. The kernel options include the radial basis function and linear kernels [45]. The choice of the cost parameter, *C*, ranged from {0.1, 1, 10, 100, 1000}. We utilized the StratifiedKFold and GridSearchCV classes in scikit-learn to perform the inner five-fold cross-validation for hyperparameter optimization and the outer five-fold cross-validation for evaluation. 

### Classification performance assessment strategy

To generate predictions from the classifiers, we fitted the training data to the model and then used the fitted model to classify samples from the testing set. For each data preprocessing combination, the datasets served as the inputs for the classification algorithm. This process determined the best-trained models to assess the internal evaluation and external independent test sets by making predictions on the tissue of origin for each sample to generate a confusion matrix. The best-trained models determined on individual folds were saved along with the associated training data scaling factors and PCA feature spaces. The test sets were scaled, when appropriate, with the previously saved training data scaling factors and then projected onto the PCA feature space of training data. The best-trained models were then applied on the transformed test set for classification. The predicted and ground truth memberships were used to derive the following classwise performance metrics for each disease class from the confusion matrix: positive predictive value, negative predictive value, accuracy, prevalence, sensitivity, specificity, false positive rate, false negative rate, area under the receiver operating characteristic (AUROC), and F1-score. The classwise performance metrics were aggregated to determine the micro-average of the AUROC and weighted F1-score, both range from 0 (worst) to 1 (best) and assess the multiclass classification model's overall performance [46]. The Shapley Additive Explanations (SHAP) values were also calculated to find the most important PCA features across all classes along with their top contributing genes [47]. A glossary of the formulas and descriptions for the classification performance metrics are provided (See Additional file 1).

### Comparison of the classifier performance results with an alternative model from the literature

To validate the results generated by our model, we used an alternative model called TULIP also trained on TCGA data to make predictions on the same independent test datasets [48]. Of the four possible convolutional neural network models created for TULIP, we used the one with all genes and 32 primary tumor types. Each of the unscaled data preprocessing combinations, previously generated for GTEx and ICGC/GEO, were separately used as an input for TULIP to generate predictions to compare to the ground truth to determine the associated weighted F1-score. We note that only unscaled versions of the data preprocessing combinations were used to make predictions on one TULIP model since scaling factors and cross-validation folds for the training set were not publicly available for the TULIP model.

### Statistics and data visualizations

Statistical analyses were performed in R using the Student's *t*-test (unpaired, one-tailed) for comparison of two groups. Each group consisted of five models evaluated from the outer folds of cross-validation. To test for significant increases in the overall performance of our machine learning classifier loaded with various datasets, the weighted F1-score based on the original datasets was compared to the 15 other modified datasets used as the input for classification. A *p* value of less than 0.05 was considered statistically significant for the Student's *t-*test. As a prerequisite, the Shapiro–Wilk test was performed (*p* value > 0.05 in all cases) for normality of the weighted F1-scores for the original dataset and modified datasets [49]. As another precondition, the Levene's test was assessed (*p* value > 0.05 in all cases) for homogeneity of variances of the weighted F1-scores for the original and modified datasets [50]. To create plots, we used the matplotlib and scikit-plot packages in Python [51, 52] as well as the ggplot2 and ggpattern packages in R [53, 54].

## Results

We quantified the effects of post-sequencing computational procedures for several combinations of normalization, batch effect adjustment, and data scaling on the RNA-Seq datasets that were used in the machine learning model building and classification process. Here, we present the findings from our analyses. Foremost, we present the best-performing combinations of data preprocessing steps, the preferred batch correction algorithm, and independent dataset used to achieve an increase in performance estimates. In addition, we demonstrate with negative results that data preprocessing tasks should not universally be applied as overall performance can be hampered in some instances. Furthermore, we identify the essential type of batch effects in this study that must be adjusted to improve classification performance.

### Limma's batch effect correction algorithm improved overall classifier performance for the independent test set of GTEx

**Fig. 2:**
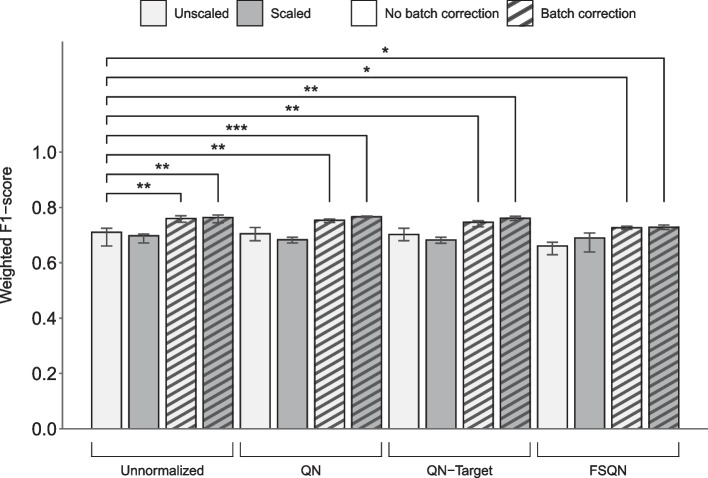
Improvement of classifier performance after Limma's batch effect correction against the GTEx test set. Weighted F1-scores as determined by SVM classifier loaded with the original dataset (left-most bar) versus the modified datasets after combinations of normalization (*Unnormalized, QN [Quantile Normalization]*, *QN-Target [Quantile Normalization with Target], FSQN [Feature-Specific Quantile Normalization])*, batch effect correction (*No batch correction, Batch correction)* and data scaling (*Unscaled, Scaled*). The training and independent test datasets were TCGA and GTEx, respectively. The batch effect correction algorithm used was Limma, and all three types of batch effect (*Protocol batch effect, Disease batch effect, and Consortium batch effect*) were adjusted. Bars indicate the median values of each group that consisted of five models evaluated from the outer folds of cross-validation. Error bars represent the 95% confidence interval. Statistical significance was determined with the Student’s *t*-test. *p < 0.05; **p < 0.01; ***p < 0.001

To determine the data preprocessing procedures that would improve overall classification performance, all 16 data preprocessing combinations based on variations of normalization, batch effect correction, and data scaling were used as inputs for the machine learning classifier to attain their associated average AUROC, weighted F1-score, and *p* value (Table 2). Data preprocessing combinations, where GTEx served as the independent test set and batch effect correction was applied, generated modified datasets. These datasets resulted in significant increase in overall performance as compared to the original datasets. The most notable increase in overall performance as compared to the original dataset (weighted F1-score = 0.71, 95% confidence interval (CI) [0.66, 0.72]) was seen with the modified dataset that underwent QN, batch effect correction, and data scaling (weighted F1-score = 0.77, *p *value = 0.0009, 95% CI [0.76, 0.77]) as seen in Fig. 2. A similar outcome was seen with the TULIP classification model applied on the same test set (See Additional file 2).

**Table 2:** Overall performance metrics of SVM classifier using data preprocessing combinations evaluated against GTEx test set related to Fig. 2

Index	Normalization	Batch effect correction	Scaling	Micro-average of AUROC	Weighted F1-score	*p* Value
1	Unnormalized	No batch correction	Unscaled	0.94 (0.93–0.95)	0.71 (0.66–0.72)	*Baseline*
2	Quantile normalization	No batch correction	Unscaled	0.93 (0.92–0.94)	0.71 (0.68–0.73)	0.2963
3	Quantile normalization with target	No batch correction	Unscaled	0.93 (0.92–0.94)	0.70 (0.68–0.72)	0.3133
4	Feature specific quantile normalization	No batch correction	Unscaled	0.92 (0.91–0.93)	0.66 (0.63–0.67)	0.9636
5	Unnormalized	Batch correction	Unscaled	0.98 (0.96–0.98)	0.76 (0.74–0.77)	0.0049**
6	Quantile normalization	Batch correction	Unscaled	0.97 (0.96–0.97)	0.75 (0.73–0.76)	0.0089**
7	Quantile normalization with target	Batch correction	Unscaled	0.97 (0.96–0.97)	0.75 (0.74–0.75)	0.0073**
8	Feature specific quantile normalization	Batch correction	Unscaled	0.96 (0.94–0.97)	0.73 (0.72–0.73)	0.0339*
9	Unnormalized	No batch correction	Scaled	0.92 (0.90–0.93)	0.70 (0.67–0.70)	0.6009
10	Quantile normalization	No batch correction	Scaled	0.90 (0.89–0.91)	0.68 (0.67–0.69)	0.7241
11	Quantile normalization with target	No batch correction	Scaled	0.89 (0.87–0.90)	0.68 (0.67–0.69)	0.7298
12	Feature specific quantile normalization	No batch correction	Scaled	0.91 (0.89–0.91)	0.69 (0.64–0.71)	0.3715
13	Unnormalized	Batch correction	Scaled	0.97 (0.96–0.98)	0.76 (0.75–0.77)	0.0026**
14	Quantile normalization	Batch correction	Scaled	0.96 (0.96–0.97)	0.77 (0.76–0.77)	0.0009***
15	Quantile normalization with target	Batch correction	Scaled	0.96 (0.96–0.97)	0.76 (0.75–0.77)	0.0016**
16	Feature specific quantile normalization	Batch correction	Scaled	0.96 (0.95–0.97)	0.73 (0.72–0.74)	0.0305*

Values indicate the median of each metric with five models evaluated from the outer folds of cross-validation; Inside the parentheses denotes the 95% confidence interval. Statistical significance was determined with the Student's *t*-test. *p < 0.05; **p < 0.01; ***p < 0.001

Visualizations of the data from the original and the best-performing modified datasets after linear projection using PCA (Figure S3), and non-linear projections using t-SNE and UMAP (Figures S4, S5) of the high-dimensional training and independent test datasets are provided.

### Limma's batch effect correction improved classwise performance of GI tissue type at the expense of other tissues

To understand the improvement in overall classification performance from original datasets as compared to the best-performing modified datasets that underwent QN, batch effect correction, and data scaling, we looked at the classwise performance of each tissue in both scenarios (Tables 3, 4). Noticeably, GI tissue type showed an enhanced F1-score in the latter datasets (F1-score = 0.59, 95% CI [0.57, 0.59]) in contrast to the former datasets (F1-score = 0.04, 95% CI [0.02, 0.04]). To a lesser extent for BRCA, there was a slight increase in F1-score seen with the latter datasets (F1-score = 0.97, 95% CI [0.95, 0.98]) over the former datasets (F1-score = 0.86, 95% CI [0.81, 0.90]). On the other hand, we observed decreases in the classwise performance metrics for HNSC (F1-score = 0.33, 95% CI [0.28, 0.48]) and PAAD (F1-score = 0.78, 95% CI [0.48, 0.89]) when using the original datasets, as compared to the overall best-performing modified datasets for HNSC (F1-score = 0.15, 95% CI [0.13, 0.19]) and PAAD (F1-score = 0.01, 95% CI [0.01, 0.01]). Further insight between the predictive models built with either the original or the best-performing modified datasets are provided by the confusion matrices, AUROC plots, and SHAP global feature importance (Figure S6, S7, S8, Table S2).

**Table 3:** Classwise performance metrics of SVM classifier using original datasets evaluated against GTEx test set

Type	n	Sensitivity	Specificity	PPV	NPV	Accuracy	AUROC	F1-score
BLCA	11	1.00 (1.00–1.00)	0.84 (0.83–0.85)	0.02 (0.02–0.02)	1.00 (1.00–1.00)	0.84 (0.83–0.85)	1.00 (1.00–1.00)	0.04 (0.04–0.04)
BRCA	304	0.79 (0.72–0.85)	1.00 (0.99–1.00)	0.94 (0.91–0.95)	0.98 (0.97–0.99)	0.98 (0.97–0.98)	1.00 (0.99–1.00)	0.86 (0.81–0.90)
CESC	6	0.50 (0.26–0.74)	0.99 (0.97–1.00)	0.06 (0.03–0.14)	1.00 (1.00–1.00)	0.99 (0.97–1.00)	0.98 (0.94–1.00)	0.09 (0.06–0.20)
COAD	281	0.63 (0.62–0.65)	1.00 (1.00–1.00)	1.00 (1.00–1.00)	0.97 (0.97–0.97)	0.97 (0.97–0.97)	0.95 (0.93–0.95)	0.78 (0.76–0.79)
GI	706	0.01 (0.00–0.02)	1.00 (1.00–1.00)	1.00 (1.00–1.00)	0.79 (0.79–0.79)	0.79 (0.79–0.79)	0.94 (0.92–0.97)	0.04 (0.02–0.04)
HNSC	101	0.77 (0.72–0.84)	0.90 (0.89–0.95)	0.21 (0.17–0.35)	0.99 (0.99–0.99)	0.90 (0.89–0.95)	0.94 (0.92–0.97)	0.33 (0.28–0.48)
KIRC	48	1.00 (0.98–1.00)	1.00 (1.00–1.00)	0.96 (0.94–1.00)	1.00 (1.00–1.00)	1.00 (1.00–1.00)	1.00 (1.00–1.00)	0.98 (0.97–0.99)
LIHC	187	1.00 (1.00–1.00)	1.00 (0.96–1.00)	1.00 (0.69–1.00)	1.00 (1.00–1.00)	1.00 (0.97–1.00)	1.00 (1.00–1.00)	1.00 (0.80–1.00)
LUAD	470	0.94 (0.88–0.98)	1.00 (1.00–1.00)	0.98 (0.97–0.99)	0.99 (0.98–1.00)	0.99 (0.98–1.00)	1.00 (1.00–1.00)	0.96 (0.93–0.98)
PAAD	263	1.00 (0.51–1.00)	0.95 (0.94–0.98)	0.64 (0.61–0.70)	1.00 (0.96–1.00)	0.95 (0.94–0.97)	1.00 (0.94–1.00)	0.78 (0.48–0.89)
PCPG	204	0.99 (0.98–0.99)	1.00 (1.00–1.00)	1.00 (1.00–1.00)	1.00 (1.00–1.00)	1.00 (1.00–1.00)	1.00 (1.00–1.00)	0.99 (0.99–1.00)
PRAD	158	0.88 (0.87–0.90)	1.00 (1.00–1.00)	1.00 (0.95–1.00)	0.99 (0.99–1.00)	0.99 (0.99–0.99)	0.99 (0.99–0.99)	0.93 (0.92–0.94)
THCA	486	0.99 (0.99–0.99)	1.00 (1.00–1.00)	1.00 (1.00–1.00)	1.00 (1.00–1.00)	1.00 (1.00–1.00)	1.00 (1.00–1.00)	0.99 (0.99–1.00)
UCEC	115	0.77 (0.52–0.95)	1.00 (1.00–1.00)	0.98 (0.95–1.00)	0.99 (0.98–1.00)	0.99 (0.98–1.00)	1.00 (1.00–1.00)	0.84 (0.65–0.98)

*n* = number of test samples; Values indicate the median of each metric with five models evaluated from the outer folds of cross-validation; Inside the parentheses denotes the 95% confidence interval

**Table 4:** Classwise performance metrics of SVM classifier using best-performing modified datasets evaluated against GTEx test set

Type	n	Sensitivity	Specificity	PPV	NPV	Accuracy	AUROC	F1-score
BLCA	11	1.00 (1.00–1.00)	0.99 (0.98–0.99)	0.22 (0.17–0.23)	1.00 (1.00–1.00)	0.99 (0.98–0.99)	1.00 (1.00–1.00)	0.35 (0.30–0.38)
BRCA	304	0.97 (0.96–0.98)	1.00 (0.99–1.00)	0.96 (0.93–0.98)	1.00 (1.00–1.00)	0.99 (0.99–1.00)	1.00 (1.00–1.00)	0.97 (0.95–0.98)
CESC	6	0.33 (0.02–0.52)	0.99 (0.98–1.00)	0.02 (0.00–0.06)	1.00 (1.00–1.00)	0.99 (0.98–0.99)	0.97 (0.96–0.99)	0.04 (0.03–0.11)
COAD	281	0.64 (0.63–0.65)	1.00 (1.00–1.00)	0.99 (0.99–0.99)	0.97 (0.97–0.97)	0.97 (0.97–0.97)	0.97 (0.97–0.98)	0.78 (0.77–0.79)
GI	706	0.71 (0.69–0.72)	0.81 (0.81–0.82)	0.50 (0.49–0.51)	0.91 (0.91–0.92)	0.79 (0.78–0.79)	0.77 (0.76–0.80)	0.59 (0.57–0.59)
HNSC	101	0.23 (0.20–0.26)	0.95 (0.95–0.96)	0.11 (0.10–0.15)	0.98 (0.97–0.98)	0.93 (0.92–0.93)	0.91 (0.91–0.93)	0.15 (0.13–0.19)
KIRC	48	0.98 (0.98–0.98)	1.00 (1.00–1.00)	1.00 (1.00–1.00)	1.00 (1.00–1.00)	1.00 (1.00–1.00)	1.00 (1.00–1.00)	0.99 (0.99–0.99)
LIHC	187	1.00 (1.00–1.00)	1.00 (1.00–1.00)	1.00 (1.00–1.00)	1.00 (1.00–1.00)	1.00 (1.00–1.00)	1.00 (1.00–1.00)	1.00 (1.00–1.00)
LUAD	470	0.98 (0.98–0.99)	1.00 (1.00–1.00)	1.00 (1.00–1.00)	1.00 (1.00–1.00)	1.00 (1.00–1.00)	1.00 (1.00–1.00)	0.99 (0.99–0.99)
PAAD	263	0.00 (0.00–0.01)	1.00 (1.00–1.00)	1.00 (1.00–1.00)	0.92 (0.92–0.92)	0.92 (0.92–0.92)	0.96 (0.92–0.98)	0.01 (0.01–0.01)
PCPG	204	0.96 (0.95–0.96)	1.00 (1.00–1.00)	1.00 (1.00–1.00)	1.00 (1.00–1.00)	1.00 (1.00–1.00)	1.00 (1.00–1.00)	0.98 (0.98–0.98)
PRAD	158	0.88 (0.86–0.89)	1.00 (1.00–1.00)	1.00 (1.00–1.00)	0.99 (0.99–0.99)	0.99 (0.99–0.99)	1.00 (1.00–1.00)	0.94 (0.92–0.94)
THCA	486	0.94 (0.93–0.95)	1.00 (1.00–1.00)	1.00 (1.00–1.00)	0.99 (0.99–0.99)	0.99 (0.99–0.99)	1.00 (1.00–1.00)	0.97 (0.96–0.97)
UCEC	115	0.78 (0.51–0.93)	1.00 (1.00–1.00)	1.00 (0.98–1.00)	0.99 (0.98–1.00)	0.99 (0.98–1.00)	1.00 (1.00–1.00)	0.88 (0.67–0.96)

*n* = number of test samples; Values indicate the median of each metric with five models evaluated from the outer folds of cross-validation; Inside the parentheses denotes the 95% confidence interval

### Limma's batch effect correction algorithm is detrimental to the overall classifier performance for the independent test set of ICGC/GEO

**Fig. 3:**
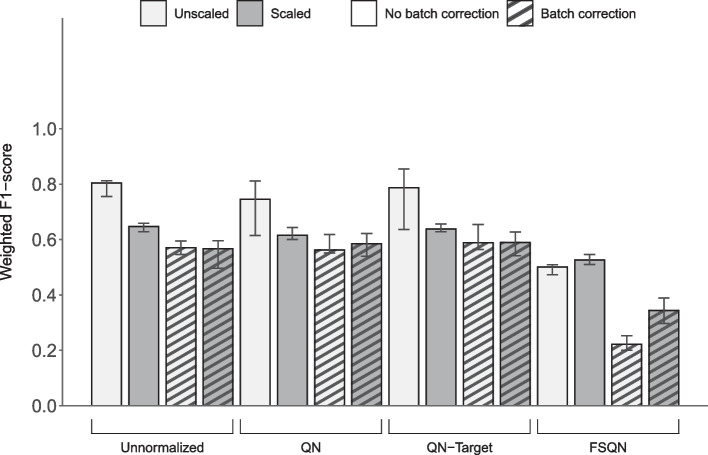
Deterioration of classifier performance after Limma's batch effect correction against the ICGC/GEO test set. Weighted F1-scores as determined by SVM classifier loaded with the original dataset (left-most bar) versus the modified datasets after combinations of normalization (*Unnormalized, QN [Quantile Normalization]*, *QN-Target [Quantile Normalization with Target], FSQN [Feature-Specific Quantile Normalization])*, batch effect correction (*No batch correction, Batch correction)* and data scaling (*Unscaled, Scaled*). The training and independent test datasets were TCGA and ICGC/GEO, respectively. The batch effect correction algorithm used was Limma, and all three types of batch effect (*Protocol batch effect, Disease batch effect, and Consortium batch effect*) were adjusted. Bars indicate the median values of each group that consisted of five models evaluated from the outer folds of cross-validation. Error bars represent the 95% confidence interval

To evaluate whether Limma's batch effect correction algorithm was effective across all datasets, we included another independent test set for comprehensive analysis. Data preprocessing combinations based on the ICGC/GEO dataset with Limma's batch effect correction applied showed reduced weighted F1-scores, as compared to when the original datasets were loaded (Fig. 3, Table 5). A similar outcome was seen with the TULIP classification model applied on the same test set (See Additional file 3).

**Table 5:** Overall performance metrics of classifier using data preprocessing combinations evaluated against ICGC/GEO test set related to Fig. 3

Index	Normalization	Batch effect correction	Scaling	Micro-average of AUROC	Weighted F1-score	*p* Value
1	Unnormalized	No batch correction	Unscaled	0.95 (0.94–0.96)	0.80 (0.76–0.81)	*Baseline*
2	Quantile normalization	No batch correction	Unscaled	0.95 (0.91–0.97)	0.75 (0.61–0.81)	0.8986
3	Quantile normalization with target	No batch correction	Unscaled	0.96 (0.92–0.97)	0.79 (0.64–0.85)	0.7418
4	Feature specific quantile normalization	No batch correction	Unscaled	0.79 (0.79–0.80)	0.50 (0.47–0.51)	1
5	Unnormalized	Batch correction	Unscaled	0.87 (0.85–0.88)	0.57 (0.55–0.60)	1
6	Quantile normalization	Batch correction	Unscaled	0.86 (0.85–0.87)	0.56 (0.55–0.62)	1
7	Quantile normalization with target	Batch correction	Unscaled	0.87 (0.85–0.87)	0.59 (0.56–0.65)	0.9999
8	Feature specific quantile normalization	Batch correction	Unscaled	0.75 (0.74–0.76)	0.22 (0.20–0.25)	1
9	Unnormalized	No batch correction	Scaled	0.94 (0.92–0.95)	0.65 (0.63–0.66)	0.8986
10	Quantile normalization	No batch correction	Scaled	0.91 (0.87–0.93)	0.62 (0.60–0.64)	1
11	Quantile normalization with target	No batch correction	Scaled	0.90 (0.86–0.93)	0.64 (0.63–0.66)	1
12	Feature specific quantile normalization	No batch correction	Scaled	0.80 (0.79–0.82)	0.53 (0.51–0.55)	1
13	Unnormalized	Batch correction	Scaled	0.84 (0.81–0.87)	0.57 (0.50–0.60)	1
14	Quantile normalization	Batch correction	Scaled	0.86 (0.85–0.87)	0.58 (0.54–0.62)	1
15	Quantile normalization with target	Batch correction	Scaled	0.87 (0.85–0.88)	0.59 (0.54–0.63)	1
16	Feature specific quantile normalization	Batch correction	Scaled	0.77 (0.77–0.77)	0.34 (0.30–0.39)	1

Values indicate the median of each metric with five models evaluated from the outer folds of cross-validation; Inside the parentheses denotes the 95% confidence interval. Statistical significance was determined with the Student's *t*-test

In attempts to gain positive changes in weighted F1-scores with the ICGC/GEO dataset, as previously shown with GTEx, we explored two alternative data preprocessing methods. First, we hypothesized that matching the tissue types between the two studies may improve the overall batch correction process. We tried preprocessing and training the machine learning model with a filtered TCGA dataset of only the six tissue types (GI, KIRC, LIHC, LUAD, PAAD, and PRAD) that were found in the ICGC/GEO dataset rather than the 14 tissues (See Additional file 4). Second, we wondered whether performing batch correction with GTEx and then applying this model to ICGC/GEO could improve the results, so we experimented with using the GTEx dataset in the batch correction step rather than ICGC/GEO dataset, while still using the latter as the independent test dataset (See Additional file 5). In both scenarios attempted, the results were similar in that no data preprocessing combinations led to an increased weighted F1-score as compared to the baseline dataset.

### Protocol and consortium batch correction had a larger impact compared to disease state batch correction.

To determine the minimum set of batch effect types that needed adjustment for improved overall performance in the current study, we examined each permutation of batch types to be corrected for. The batch effect correction algorithm used was Limma and the parameters of *Unnormalized* and *Scaled* were fixed. Batch effect corrections specifically for protocol and consortium batch types were essential to provide a significant improvement in weighted F1-scores. In this context, adjusting for the disease batch effect was not required, but did yield further improvement in the weighted F1-score (Figure S9). Classwise performance metrics for the adjustments of the batch types in the combinations that led to enhanced overall classifier predictive power are provided for protocol and consortium batch effects (See Additional file 6) and for protocol, disease, and consortium batch effects (See Additional file 7).

### Limma batch correction results in better prediction compared to ComBat

To find a batch correction algorithm that enhanced classifier performance, a few popular alternatives from the literature were compared. With Limma, all data preprocessing combinations that included batch effect correction showed an increase in weighted F1-scores regardless of normalization or data scaling procedures (Fig. 2). The default ComBat method led to an increase in weighted F1-scores whenever batch effect correction was applied in cases of *Unnormalized* and *FSQN* (Figure S10). With reference-batch ComBat, we also see these increases in weighted F1-scores, except where the data preprocessing combination was *Unscaled* and *Unnormalized* (Figure S11)*.* Although all three resulted in improved performance, Limma's batch effect correction algorithm led to better overall classification performance than either the default usage of ComBat or reference-batch ComBat.

## Discussion

The current comparison study demonstrates that the application of data preprocessing operations has varied effects on classifier performance for the molecular classification of tissue types with RNA-Seq data. Whether the effect is beneficial or adverse depends on the specific datasets used. In our study design where TCGA samples served as the training set, we explored various data preprocessing procedures, including—normalization, batch effect correction, and data scaling. The constructed classification models were then compared based on their overall performance on independent samples generated from separate consortia, GTEx and ICGC/GEO (Fig. 1).

There have been numerous studies on tissue classification using gene expression data, but studies that test the class predictor across independent datasets are not as common [55]. Typically, the classifier is trained on a subset of samples from a study and tested on the remaining portion of the unseen samples from the same pool [56–58]. Having samples from the same project in the training and test sets can potentially lead to over-optimistic measures of performance due to overfitting, where the model learns the noise of the training set, and in this case detects the same noise in the test set [59]. In studies where the test data are truly independent, with none of the test samples from the same study as the training dataset, it is generally expected to see lower classification performance for these independent test samples as compared to when the test samples came from the same pool as the training samples [60, 61]. The gold standard for demonstration of the power of gene expression classification is to test against independent datasets to provide an unbiased learning performance estimate [62]. Therefore, our current study emphasized the use of a couple sources of independent samples for testing. The performance estimated from an independent dataset informs us about the generalizability of our model, how useful the classifier will be on unseen test data, which is an important consideration for adaptation into the clinical setting [63].

Batch effect correction was a sufficient data preprocessing task to gain an improvement in classification performance when TCGA and GTEx were used as the training and testing set, respectively (Fig. 2). In contrast, none of the data preprocessing techniques helped increase overall performance when ICGC/GEO served as the test set (Fig. 3), warning other researchers to exercise caution when considering data preprocessing procedures as part of their classification pipeline. We note that there is a possibility of information leakage during batch effect adjustment of the combined datasets during data preprocessing (Figure S2). This can lead to over-estimated performance when the correction transfers information from the test data to the training process [12]. However, we also explored the reference-batch ComBat method, which keeps the training and test datasets separate during batch correction. The performance measures on reference-batch ComBat were slightly lower, yet there were still improvements after batch correction (Figure S11). So, we can say that the overestimation is minor, and that batch correction is still recommended when GTEx serves as the independent test set.

Among the batch effect correction methods, the basic linear estimation of batch effect by Limma showed better performance compared to the shrinkage estimator provided by ComBat [35, 64]. Although initially surprised by this result, we realized this may be explained by our sample size where we have at least 100 samples per class and at least several hundreds of samples per batch for each batch group. With this sample size, the bias–variance trade-off of shrinkage provided by ComBat may be unnecessary and may actually hinder the estimation of batch effect.

While we achieved positive results when testing against GTEx, by contrast negative results were obtained when ICGC/GEO served as the independent test set (Figs. 2, 3). We further validated these results with an alternative model from the literature called TULIP, a convolution neural network-based classifier, also trained with TCGA data [48]. Predictions made by the TULIP model seemed to show an improvement in weighted F1-scores with GTEx as the test dataset (See Additional file 2) and a deterioration of the overall classification performance metrics when ICGC/GEO served as the independent test set (See Additional file 3). These outcomes appear to be consistent with the results from our SVM-based classifier. Notably, the weighted F1-scores achieved by our SVM model appeared to be better than the overall performance estimates attained by the TULIP model. This scenario where we have elevated performance scores on one test dataset that is offset by lower performance metrics against another dataset is not uncommon in the literature. The "no free lunch theorem" implies that no method is expected to work well with all datasets [65].

We attempted alternate approaches to obtain a positive result with ICGC/GEO as the independent test set by modifying our classification pipeline, such as training the classifier with a limited set of tissues from TCGA, to match what is available in the ICGC/GEO dataset (see Additional file 4), and also performed batch effect correction with information from the GTEx dataset (see Additional file 5), both to no avail. Since our ICGC/GEO dataset is severely imbalanced, one reason for these negative results could be that batch effect correction on imbalance datasets may also cause false differences leading to undesired consequences and misinterpretation [66]. Another reason for this discrepancy could be due to predictor measurement heterogeneity since the gene expression values are sourced from separate individual studies [67]. Previously, Ellis et al*.* [20] built a predictor for tissue types trained with the GTEx data and then tested against independent test sets of aggregated Sequence Read Archive studies along with a TCGA dataset, and similarly attained a much lower overall performance score (51.9%) for the aggregated dataset as compared to the performance estimate (76.8%) on the TCGA data. There would be more technical variation expected when there are pronounced differences in protocols and reagents used to generate the RNA-Seq datasets. Ideally, the same strategy should be employed during procurement of samples so that there is a homogeneity of predictor measurements; however, this is difficult with studies done independently across multiple labs. Considering that classifying the tissue-of-origin is likely one of the easiest problems for RNA-Seq sample classifications, with signals that tend to be stronger as compared to other treatments or groupings, this seems to indicate that the possibility of aggregating disparate data from multiple sources for the purpose of machine learning-based predictions has a rather pessimistic prospect.

Data scaling has previously been reported to generally help learning models achieve better performance estimates [68]. However, this was not the case seen in our results, as weighted F1-scores with scaled datasets were mostly maintained or in some cases made worse (Figs. 2, 3). Only the data preprocessing combination with FSQN and batch correction applied that used ICCG/GEO as the independent test dataset did data scaling show a better weighted F1-score than its unscaled counterpart. Similarly, normalization did not have a noticeable effect on the constructed classifiers to improve performance estimates. In one data preprocessing combination, where the normalization type applied was FSQN and ICGC/GEO served as the independent test set, the weighted F1-score was markedly reduced. This is likely because the features, read counts for each gene, in an RNA-Seq dataset is in essence already an intrinsically scaled measurement, considering that the counts are compositional counts of a predefined library size. Also, all the features are quantified in the same unit (i.e., read counts), and although there are large magnitude differences between the genes, each feature measurement reflects the true expression difference rather than the difference in unit or possible range.

This work has several limitations to note. Foremost, we utilized imbalanced datasets with regards to both training and testing sets. This bias in the training dataset can influence the machine learning algorithm with less predictive power for the less represented classes. Some approaches to balance the datasets are random oversampling, which duplicates examples from the minority classes, and undersampling, where some samples are excluded. In addition, the ICGC/GEO independent dataset is relatively small and does not contain all the tissues as the TCGA training nor GTEx test datasets. A dataset with more samples and representing more types of cancers could serve as another independent test for more robust classification results. Furthermore, a constraint of the proposed model is that batches are assumed to be known, enabling methods to correct for batches [9, 22, 35]. There are also alternative approaches that aim to estimate the unknown latent variables [10, 69, 70]. A large-scale transcriptomic study, such as GTEx, was able to find latent variables in the data that are correlated with specific environment and conditions [12]. Assuming there are a limited number of systematic variables that can affect the data, it will be worthwhile to get a better understanding of the effects of different kinds of latent variables on transcript quantification, so we can recommend the appropriate data preprocessing methods depending on the estimated latent variables. There has been progress made in machine learning to understand the problem of dataset shift and approaches to solve the issue. We could try applying classical methods based on covariate shift adaptation to this particular problem [71, 72]. If enough information is available on the latent variables that affect the data, then we could even attempt to generate synthetic datasets with the problematic latent effects and attempt to fit a model that is able to correct the synthetic effects using generative adversarial networks [73–75].

Another future direction that can be considered is to explore if changes in how the RNA-Seq datasets are generated will affect the downstream classification results. The term "data preprocessing" can refer to different aspects of a RNA-Seq analysis workflow. Data preprocessing can refer to either quantification, the process from raw sequencing reads to gene counts, or part of a classification task, which takes place after the counts data are generated. While the current study focuses on the latter, the former can also be investigated. For example, *recount2* provides an alternate version of the publicly deposited expression data that have been summarized with a single quantification pipeline [16]. While the starting point for our research was the counts sourced from the original consortia, other investigators can try the *recount2* version of the counts data. They could even begin their analyses with raw reads or sequence alignment maps and evaluate the impact of other types of data preprocessing procedures on classification performance.

## Conclusion

Here, we compared 16 data preprocessing combinations based on variations of normalization, batch effect correction, and data scaling in efforts to enhance a machine learning model to correctly predict cancer tissue types for more unseen samples. We utilized publicly available large-scale RNA-Seq datasets to construct classifiers to resolve tissue of origin against an independent test set. Our analyses demonstrate that the overall classification performance measured by weighted F1-score on a model trained with TCGA data and tested against the GTEx dataset, can be improved by applying batch effect correction. On the other hand, when ICGC/GEO served as the test set, the investigations showed the overall performance metrics ended up being maintained or declined. It seems that normalization and data scaling have limited utility in improving the performance estimates, at least in the current context. There was no universal combination of data preprocessing techniques that improved the overall classification performance against all independent test datasets, highlighting the challenges of generalizing machine learning classifiers.

### Supplementary Information

Additional file 1.

Additional file 2. 

Additional file 3. 

Additional file 4. 

Additional file 5. 

Additional file 6.

Additional file 7. 

Additional file 8. 

Additional file 9. 

## Data Availability

The raw data used in this study were obtained from the TCGA database (https://portal.gdc.cancer.gov/), the GTEx database (https://gtexportal.org/home/), the ICGC database (https://dcc.icgc.org/), and the GEO database (https://www.ncbi.nlm.nih.gov/geo/). The aggregated datasets used and/or analyzed during the current study are available from the corresponding author on reasonable request. The source code is available in the GitHub repository: https://github.com/HanLabUNLV/transcriptomic_predictions.

## Abbreviations

AUROC: Area under the receiver operating characteristic
BCGSC: British Columbia Genome Sequencing Center
CI: Confidence interval
FSQN: Feature Specific Quantile Normalization
GEO: Gene Expression Omnibus
GTEx: Genotype-Tissue Expression
ICGC: International Cancer Genome Consortium
QN: Quantile normalization
QN-Target: Quantile normalization with target
RNA-Seq: RNA sequencing
SHAP: SHapley Additive exPlanations
SVM: Support vector machine
TCGA: The Cancer Genome Atlas
UNC: University of North Carolina
