# Supplementary material for: A comparison of RNA-Seq data preprocessing pipelines for transcriptomic predictions across independent studies
Source: BMC Bioinformatics. 2024 May 8;25:181. doi: 10.1186/s12859-024-05801-x (PMC11080237; doi:10.1186/s12859-024-05801-x)
Supplement: Supplementary file 1 — Additional file 1. [file 12859_2024_5801_MOESM1_ESM.docx]

| **Table A1. Glossary of classification performance metrics** | | |
| --- | --- | --- |
|  | **Formula** | **Description** |
| **True Positive** (**TP**) also called hits |  | the number of samples that are correctly predicted the positive class |
| **False Negative** (**FN**) also called Type II error or misses |  | the number of samples that are incorrectly predicted the negative class |
| **False Positive** (**FP**) also called Type I error or false alarms |  | the number of samples that are incorrectly predicted the positive class |
| **True Negative** (**TN**) |  | the number of samples that are correctly predicted the negative class |
| **Sensitivity** is also known as **True Positive Rate (TPR)** or **Recall** | $Sensitivity=\frac{TP}{TP+FN}$ | the probability of the model to make a positive prediction for the entire group of positive observations |
| **Specificity** is also known as the **True Negative Rate** (**TNR**) | $Specificity=\frac{TN}{TN+FP}$ | the probability of the model to make a negative prediction for the entire group of negative observations |
| **Positive Predictive Value** (**PPV**) is also known as **Precision** | $PPV=\frac{TP}{TP+FP}$ | the proportion of correct positive predictions for the total number of positive observations |
| **Negative Predictive Value** (**NPV**) | $NPV=\frac{TN}{TN+FN}$ | the proportion of correct negative predictions for the total number of negative observations |
| **Accuracy (ACC)** | $ACC=\frac{TP + TN}{TP+TN+FP+FN}$ | the proportion of correct predictions |
| **Area under the receiver operating characteristic (AUROC)** |  | a measurement of a model's ability to discriminate between different classes |
| **F1-score** is also known as **F-score** and **F1** | *F1-score* $=2\times\frac{Precision \times Recall}{Precision + Recall}$ | the harmonic mean of precision and recall |
| **Micro-average of AUROC** |  | the overall discriminative performance of a model across multiple classes, by consider the total true positives and false positives across all classes |
| **Weighted F1-score** | $Weighted F1\text{-}score=\sum_{i=1}^{N} w_{i} \times{F1\text{-}score}_{i}$ | the weighted mean of F1-score with weights equal to class probability |
