# Supplementary material for: A comparison of RNA-Seq data preprocessing pipelines for transcriptomic predictions across independent studies
Source: BMC Bioinformatics. 2024 May 8;25:181. doi: 10.1186/s12859-024-05801-x (PMC11080237; doi:10.1186/s12859-024-05801-x)
Supplement: Supplementary file 3 — Additional file 3. [file 12859_2024_5801_MOESM3_ESM.docx]

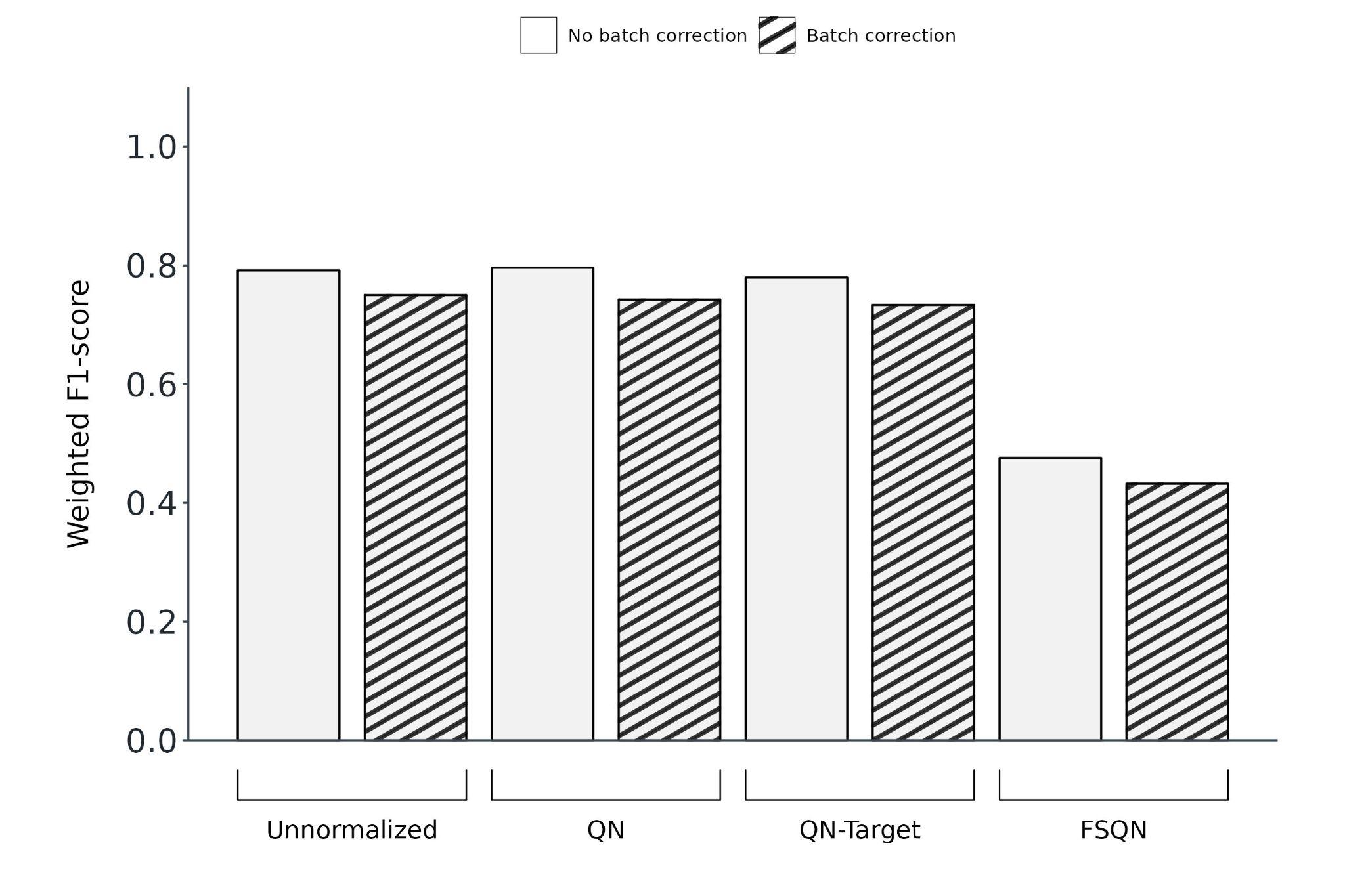


**[Figure A3] TULIP classifier performance against the ICGC/GEO test set.** Weighted F1-scores as determined by TULIP's convoluted neural network classifier tested with the original dataset (left-most bar) versus the modified datasets after combinations of normalization (*Unnormalized, QN [Quantile Normalization]*, *QN-Target [Quantile Normalization with Target], FSQN [Feature-Specific Quantile Normalization])*, and batch effect correction (*No batch correction, Batch correction)*. All datasets were unscaled. The batch effect correction algorithm used was Limma and all three types of batches (*Protocol batch effect, Disease batch effect, and Consortium batch effect*) were adjusted.
