# Supplementary material for: A comparison of RNA-Seq data preprocessing pipelines for transcriptomic predictions across independent studies
Source: BMC Bioinformatics. 2024 May 8;25:181. doi: 10.1186/s12859-024-05801-x (PMC11080237; doi:10.1186/s12859-024-05801-x)
Supplement: Supplementary file 4 — Additional file 4. [file 12859_2024_5801_MOESM4_ESM.docx]

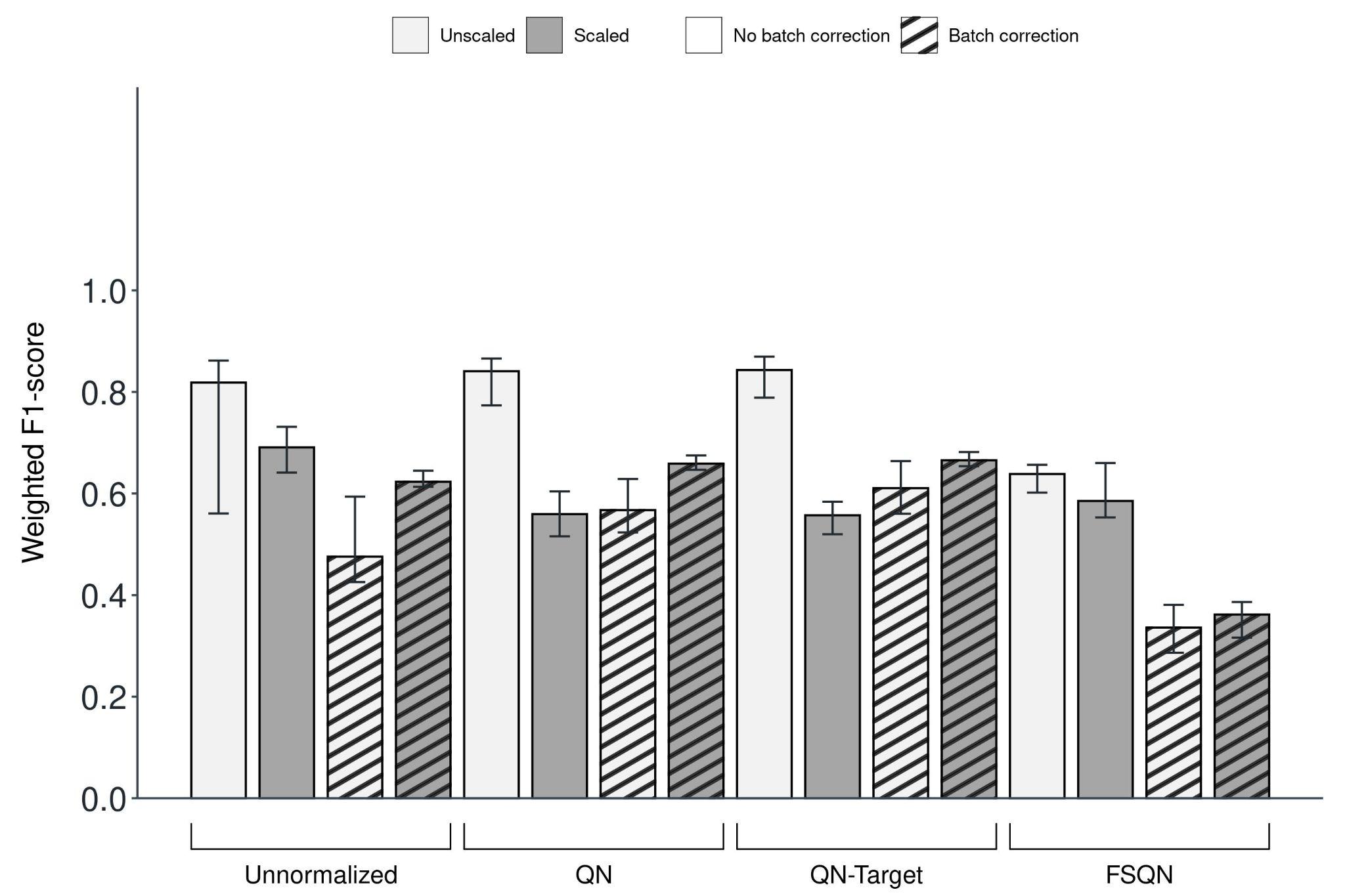


**[Figure A4] Poor classifier performance after Limma's batch effect correction against the ICGC/GEO test set with filtered tissue types.** Weighted F1-scores as determined by SVM classifier loaded with the original dataset (left-most bar) versus the modified datasets after combinations of normalization (*Unnormalized, QN [Quantile Normalization]*, *QN-Target [Quantile Normalization with Target], FSQN [Feature-Specific Quantile Normalization])*, batch effect correction (*No batch correction, Batch correction)* and data scaling (*Unscaled, Scaled*). The training and independent test datasets were TCGA filtered to the six tissue types (GI, KIRC, LIHC, LUAD, PAAD, and PRAD) and ICGC/GEO, respectively. The batch effect correction algorithm used was Limma and all three types of batches (*Protocol batch effect, Disease batch effect, and Consortium batch effect*) were adjusted. Bars indicate the median values of each group that consisted of five models evaluated from the outer folds of cross-validation. Error bars represent the 95% confidence interval.
