# Supplementary material for: A comparison of RNA-Seq data preprocessing pipelines for transcriptomic predictions across independent studies
Source: BMC Bioinformatics. 2024 May 8;25:181. doi: 10.1186/s12859-024-05801-x (PMC11080237; doi:10.1186/s12859-024-05801-x)
Supplement: Supplementary file 6 — Additional file 6. [file 12859_2024_5801_MOESM6_ESM.docx]

| **Table A6. Classwise performance metrics for batch effect correction of protocol and consortium batch types related to Figure S9** | | | | | | | | | |
| --- | --- | --- | --- | --- | --- | --- | --- | --- | --- |
| **Type** | ***n*** | **Sensitivity** | **Specificity** | **PPV** | **NPV** | **Accuracy** | **AUROC** | **F1-score** |  |
| BLCA | 11 | 1.00 (1.00-1.00) | 0.96 (0.95-0.96) | 0.07 (0.06-0.07) | 1.00 (1.00-1.00) | 0.96 (0.95-0.96) | 1.00 (1.00-1.00) | 0.13 (0.12-0.14) |  |
| BRCA | 304 | 0.96 (0.96-0.98) | 0.99 (0.99-0.99) | 0.91 (0.89-0.93) | 1.00 (1.00-1.00) | 0.99 (0.99-0.99) | 1.00 (1.00-1.00) | 0.94 (0.93-0.95) |  |
| CESC | 6 | 0.33 (0.18-0.42) | 0.99 (0.98-1.00) | 0.06 (0.02-0.09) | 1.00 (1.00-1.00) | 0.99 (0.98-0.99) | 0.99 (0.94-1.00) | 0.10 (0.04-0.14) |  |
| COAD | 281 | 0.66 (0.65-0.66) | 1.00 (1.00-1.00) | 0.98 (0.97-0.99) | 0.97 (0.97-0.97) | 0.97 (0.97-0.97) | 0.98 (0.97-0.98) | 0.79 (0.78-0.79) |  |
| GI | 706 | 0.86 (0.85-0.86) | 0.86 (0.85-0.86) | 0.42 (0.41-0.43) | 0.84 (0.84-0.85) | 0.76 (0.75-0.76) | 0.75 (0.72-0.76) | 0.40 (0.39-0.42) |  |
| HNSC | 101 | 0.52 (0.51-0.61) | 0.91 (0.90-0.92) | 0.98 (0.98-0.99) | 0.98 (0.98-0.99) | 0.90 (0.89-0.91) | 0.92 (0.91-0.93) | 0.24 (0.22-0.29) |  |
| KIRC | 48 | 0.98 (0.98-0.99) | 1.00 (1.00-1.00) | 1.00 (0.93-1.00) | 1.00 (1.00-1.00) | 1.00 (1.00-1.00) | 1.00 (1.00-1.00) | 0.99 (0.96-1.00) |  |
| LIHC | 187 | 0.98 (0.98-1.00) | 1.00 (1.00-1.00) | 1.00 (1.00-1.00) | 1.00 (1.00-1.00) | 1.00 (1.00-1.00) | 1.00 (1.00-1.00) | 0.99 (0.99-1.00) |  |
| LUAD | 470 | 0.99 (0.99-1.00) | 1.00 (1.00-1.00) | 1.00 (0.99-1.00) | 1.00 (1.00-1.00) | 1.00 (1.00-1.00) | 1.00 (1.00-1.00) | 0.99 (0.99-1.00) |  |
| PAAD | 263 | 0.01 (-0.01-0.09) | 1.00 (1.00-1.00) | 1.00 (0.98-1.00) | 0.92 (0.92-0.93) | 0.92 (0.92-0.93) | 0.99 (0.79-1.00) | 0.02 (0.02-0.17) |  |
| PCPG | 204 | 0.90 (0.83-0.93) | 1.00 (1.00-1.00) | 1.00 (1.00-1.00) | 0.99 (0.99-1.00) | 0.99 (0.99-1.00) | 1.00 (1.00-1.00) | 0.95 (0.91-0.97) |  |
| PRAD | 158 | 0.87 (0.86-0.88) | 1.00 (1.00-1.00) | 1.00 (1.00-1.00) | 0.99 (0.99-0.99) | 0.99 (0.99-0.99) | 0.99 (0.99-1.00) | 0.93 (0.93-0.93) |  |
| THCA | 486 | 0.98 (0.97-0.98) | 1.00 (1.00-1.00) | 1.00 (1.00-1.00) | 1.00 (1.00-1.00) | 1.00 (1.00-1.00) | 1.00 (1.00-1.00) | 0.99 (0.99-0.99) |  |
| UCEC | 115 | 0.96 (0.93-0.97) | 1.00 (1.00-1.00) | 0.97 (0.96-0.97) | 1.00 (1.00-1.00) | 1.00 (1.00-1.00) | 1.00 (1.00-1.00) | 0.96 (0.94-0.97) |  |
| *n* = number of test samples; Values indicate the median of each metric with five models evaluated from the outer folds of cross-validation; Inside the parentheses denotes the 95% confidence interval. | | | | | | | | | |
|  | | | | | | | | | |
