# Supplementary material for: A comparison of RNA-Seq data preprocessing pipelines for transcriptomic predictions across independent studies
Source: BMC Bioinformatics. 2024 May 8;25:181. doi: 10.1186/s12859-024-05801-x (PMC11080237; doi:10.1186/s12859-024-05801-x)
Supplement: Supplementary file 7 — Additional file 7. [file 12859_2024_5801_MOESM7_ESM.docx]

| **Table A7. Classwise performance metrics for batch effect correction of protocol, disease, and consortium batch types related to Figure S9** | | | | | | | | |
| --- | --- | --- | --- | --- | --- | --- | --- | --- |
| **Type** | ***n*** | **Sensitivity** | **Specificity** | **PPV** | **NPV** | **Accuracy** | **AUROC** | **F1-score** |
| BLCA | 11 | 1.00 (1.00-1.00) | 0.97 (0.97-0.98) | 0.10 (0.08-0.13) | 1.00 (1.00-1.00) | 0.97 (0.97-0.98) | 1.00 (1.00-1.00) | 0.18 (0.16-0.23) |
| BRCA | 304 | 0.95 (0.93-0.97) | 0.99 (0.98-0.99) | 0.89 (0.84-0.90) | 1.00 (0.99-1.00) | 0.98 (0.98-0.99) | 1.00 (1.00-1.00) | 0.92 (0.88-0.93) |
| CESC | 6 | 0.67 (0.20-0.80) | 0.98 (0.97-0.99) | 0.05 (0.01-0.09) | 1.00 (1.00-1.00) | 0.98 (0.97-0.99) | 0.98 (0.95-0.99) | 0.11 (0.04-0.17) |
| COAD | 281 | 0.65 (0.64-0.66) | 1.00 (1.00-1.00) | 0.98 (0.98-0.99) | 0.97 (0.97-0.97) | 0.97 (0.97-0.97) | 0.97 (0.96-0.98) | 0.79 (0.78-0.79) |
| GI | 706 | 0.55 (0.50-0.58) | 0.85 (0.84-0.86) | 0.50 (0.47-0.51) | 0.88 (0.86-0.88) | 0.79 (0.78-0.79) | 0.79 (0.78-0.82) | 0.53 (0.49-0.54) |
| HNSC | 101 | 0.38 (0.25-0.42) | 0.93 (0.92-0.94) | 0.12 (0.10-0.15) | 0.98 (0.98-0.98) | 0.91 (0.90-0.92) | 0.90 (0.90-0.91) | 0.18 (0.15-0.22) |
| KIRC | 48 | 0.98 (0.96-0.98) | 1.00 (1.00-1.00) | 1.00 (1.00-1.00) | 1.00 (1.00-1.00) | 1.00 (1.00-1.00) | 1.00 (1.00-1.00) | 0.99 (0.98-0.99) |
| LIHC | 187 | 0.99 (0.99-1.00) | 1.00 (1.00-1.00) | 1.00 (1.00-1.00) | 1.00 (1.00-1.00) | 1.00 (1.00-1.00) | 1.00 (1.00-1.00) | 1.00 (1.00-1.00) |
| LUAD | 470 | 1.00 (0.99-1.00) | 1.00 (1.00-1.00) | 1.00 (1.00-1.00) | 1.00 (1.00-1.00) | 1.00 (1.00-1.00) | 1.00 (1.00-1.00) | 1.00 (0.99-1.00) |
| PAAD | 263 | 0.04 (0.01-0.09) | 1.00 (1.00-1.00) | 1.00 (0.90-1.00) | 0.92 (0.92-0.93) | 0.92 (0.92-0.93) | 1.00 (0.86-1.00) | 0.07 (0.02-0.16) |
| PCPG | 204 | 0.97 (0.96-0.99) | 1.00 (1.00-1.00) | 1.00 (1.00-1.00) | 1.00 (1.00-1.00) | 1.00 (1.00-1.00) | 1.00 (1.00-1.00) | 0.99 (0.98-0.99) |
| PRAD | 158 | 0.87 (0.86-0.88) | 1.00 (1.00-1.00) | 1.00 (1.00-1.00) | 0.99 (0.99-0.99) | 0.99 (0.99-0.99) | 0.99 (0.99-1.00) | 0.93 (0.93-0.93) |
| THCA | 486 | 0.97 (0.96-0.97) | 1.00 (1.00-1.00) | 1.00 (1.00-1.00) | 1.00 (0.99-1.00) | 1.00 (0.99-1.00) | 1.00 (1.00-1.00) | 0.99 (0.98-0.99) |
| UCEC | 115 | 0.92 (0.64-0.98) | 1.00 (1.00-1.00) | 0.98 (0.97-1.00) | 1.00 (0.99-1.00) | 1.00 (0.99-1.00) | 1.00 (1.00-1.00) | 0.95 (0.78-0.98) |
| *n* = number of test samples; Values indicate the median of each metric with five models evaluated from the outer folds of cross-validation; Inside the parentheses denotes the 95% confidence interval. | | | | | | | | |
|  | | | | | | | | |
