# Supplementary material for: A comparison of RNA-Seq data preprocessing pipelines for transcriptomic predictions across independent studies
Source: BMC Bioinformatics. 2024 May 8;25:181. doi: 10.1186/s12859-024-05801-x (PMC11080237; doi:10.1186/s12859-024-05801-x)
Supplement: Supplementary file 8 — Additional file 8. [file 12859_2024_5801_MOESM8_ESM.docx]

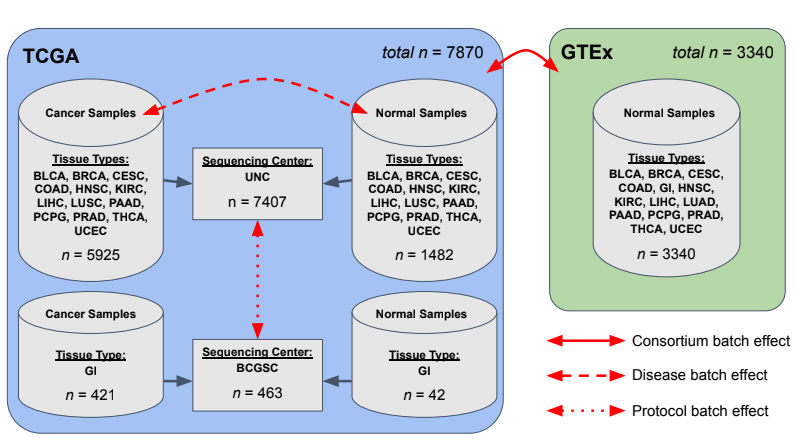


**[Figure S1] Types of batch effects present in this study.** The variations of batch effects that were identified in the current study include the sources of the genome-wide expression datasets (*Consortium batch effect*), the presence or absence of cancer (*Disease batch effect*), and the choice of sequencing center (*Protocol batch effect*). These are indicated by solid, dashed, and dotted red double-arrow headed lines, respectively. The different tissue types associated and the number of samples, *n*, are also specified.


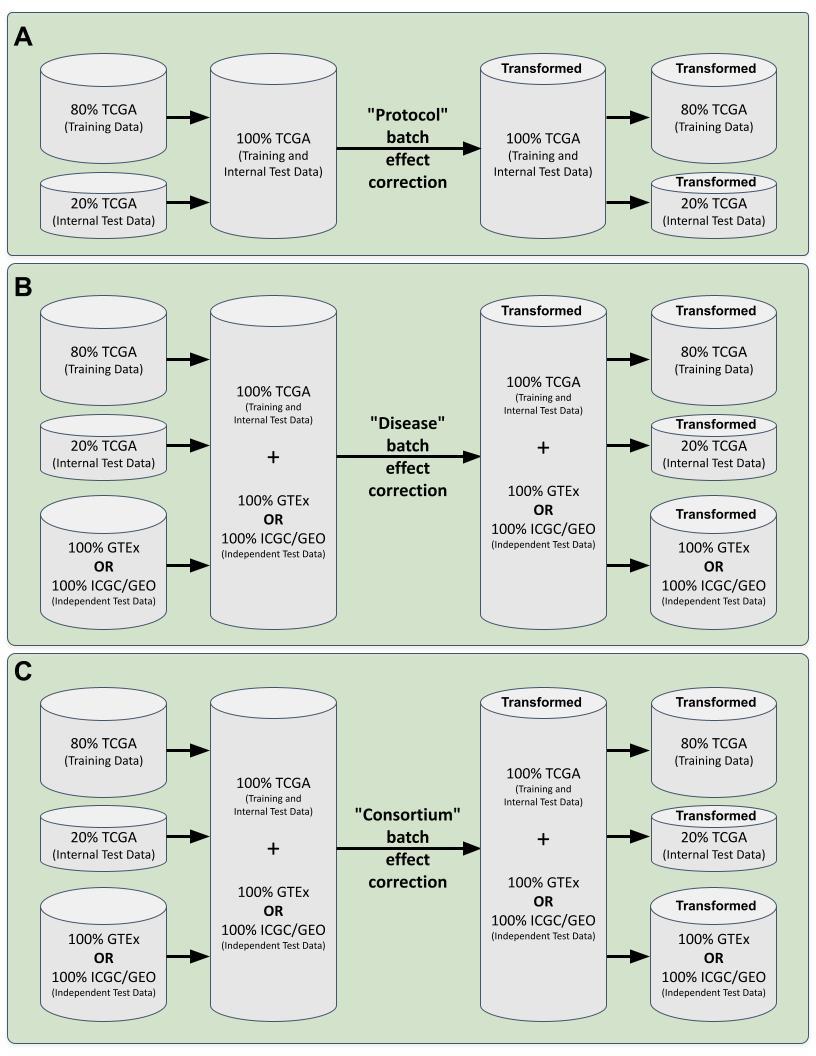


**[Figure S2] Batch effect correction approaches for each type of batch effect.** The general procedure for adjusting each type of batch effect type was to combine the relevant datasets into a merged dataset. The batch effect correction algorithm was then applied to this merged dataset, followed by splitting up the transformed merged dataset back into their respective original datasets. For the *Protocol batch effect* (**A**), only the training data, comprising 80% TCGA data and the internal test data, consisting 20% of TCGA data were combined. Conversely, for both the *Disease batch effect* (**B**) and *Consortium batch effect* (**C**), the combination included the training data (80% of TCGA), internal test data (20% TCGA of TCGA), and 100% of the independent test set (either GTEx or ICGC/GEO).


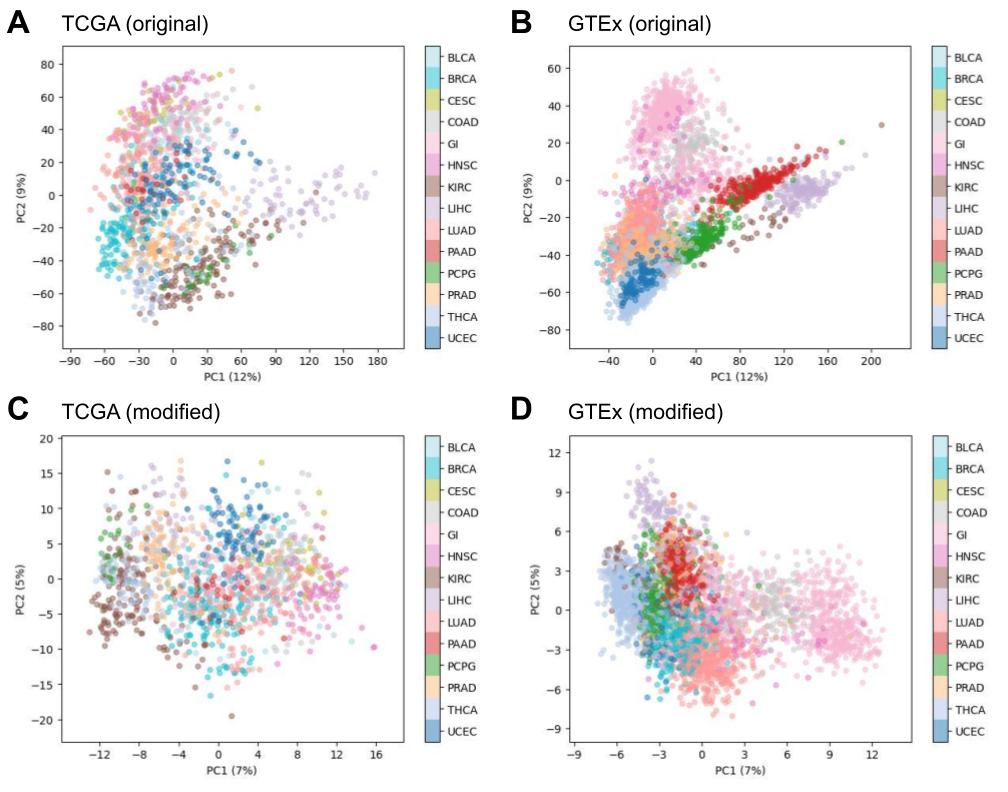


**[Figure S3] Principal component analyses of the RNA-Seq data.** Principal component analysis was performed on the RNA-Seq data of the original datasets (**A**, **B |** *Unnormalized, No batch correction, Unscaled***)** and the best-performing modified dataset (**C**, **D** | *QN, Batch correction, Scaled*). The training data (**A**, **C**) were from TCGA and the independent test data (**B**, **D**) were from GTEx. The batch effect correction algorithm used was Limma and all three types of batches (*Protocol batch effect, Disease batch effect, and Consortium batch effect*) were adjusted. Each dot represents a sample and its color indicates the tissue type. Indicated inside the parenthesis is the percentage of variance explained by the corresponding principal component (PC). Along the x-axis is PC1 and along the y-axis is PC2. These plots were taken from one of the models evaluated from the outer folds of cross-validation.


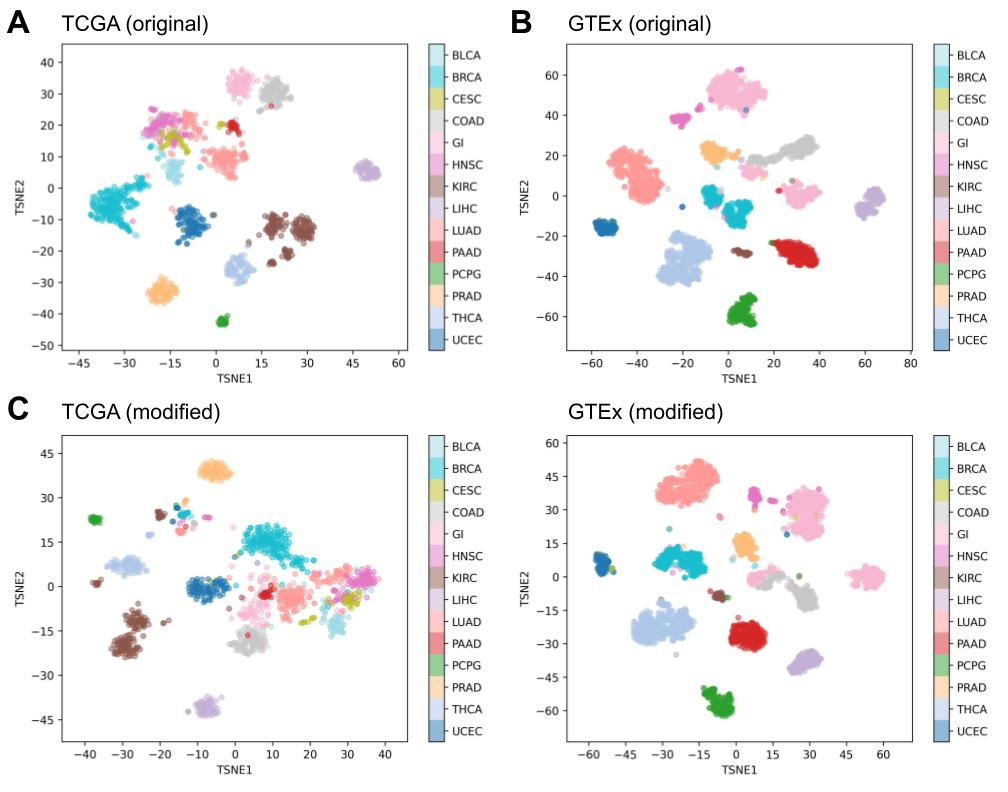


**[Figure S4] t-Distributed stochastic neighbor embedding of the RNA-Seq data.** t-Distributed stochastic neighbor embedding was performed on the RNA-Seq data of the original datasets (**A**, **B** | *Unnormalized, No batch correction, Unscaled*) and the best-performing modified dataset (**C**, **D** | *QN, Batch correction, Scaled*). The training data (**A**, **C**) were from TCGA and the independent test data (**B**, **D**) were from GTEx. The batch effect correction algorithm used was Limma and all three types of batches (*Protocol batch effect, Disease batch effect, and Consortium batch effect*) were adjusted. Each dot represents a sample and its color indicates the tissue type. These plots were taken from one of the models evaluated from the outer folds of cross-validation.


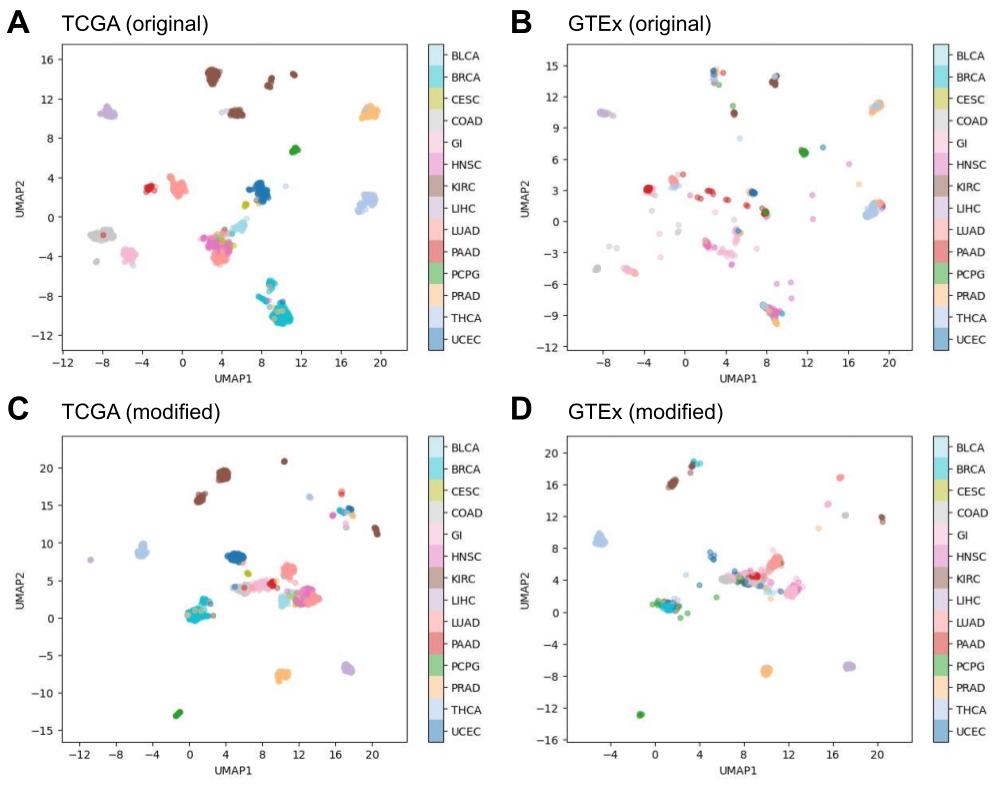


**[Figure S5] Uniform manifold approximation and projection of the RNA-Seq data.** Uniform manifold approximation was performed on the RNA-Seq data of the original datasets (**A**, **B |** *Unnormalized, No batch correction, Unscaled***)** and the best-performing modified dataset (**C**, **D** | *QN, Batch correction, Scaled*). The training data (**A**, **C**) were from TCGA and the independent test data (**B**, **D**) were from GTEx. The batch effect correction algorithm used was Limma and all three types of batches (*Protocol batch effect, Disease batch effect, and Consortium batch effect*) were adjusted. Each dot represents a sample and its color indicates the tissue type. These plots were taken from one of the models evaluated from the outer folds of cross-validation.


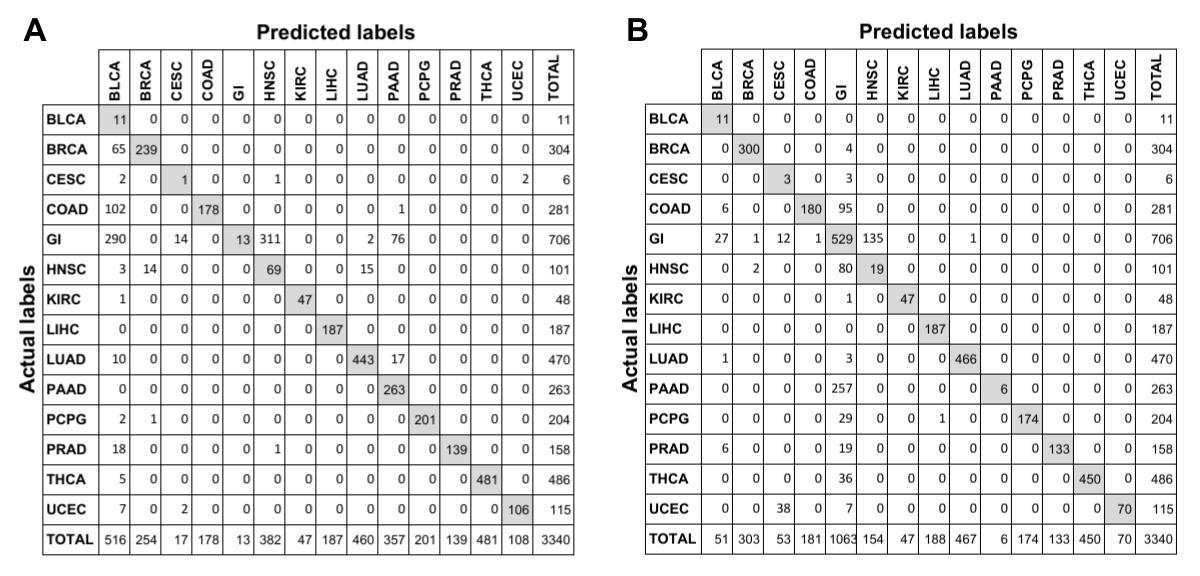


**[Figure S6] Comparison of confusion matrices by tissue types between original and best-performing datasets.**  The confusion matrices show the relationship between each multiclass classifier prediction *versus* the ground truth labels after the classifiers were loaded with either the original datasets (**A** | *Unnormalized, No batch correction, Unscaled*) or the best-performing modified datasets (**B** | *QN, Batch correction, Scaled*). The training and independent test datasets were TCGA and GTEx, respectively. The batch effect correction algorithm used was Limma and all three types of batches (*Protocol batch effect, Disease batch effect, and Consortium batch effect*) were adjusted. These confusion matrices are taken from one of the models evaluated from the outer folds of cross-validation. Values in the diagonal (gray cells) correspond to correct classifications.


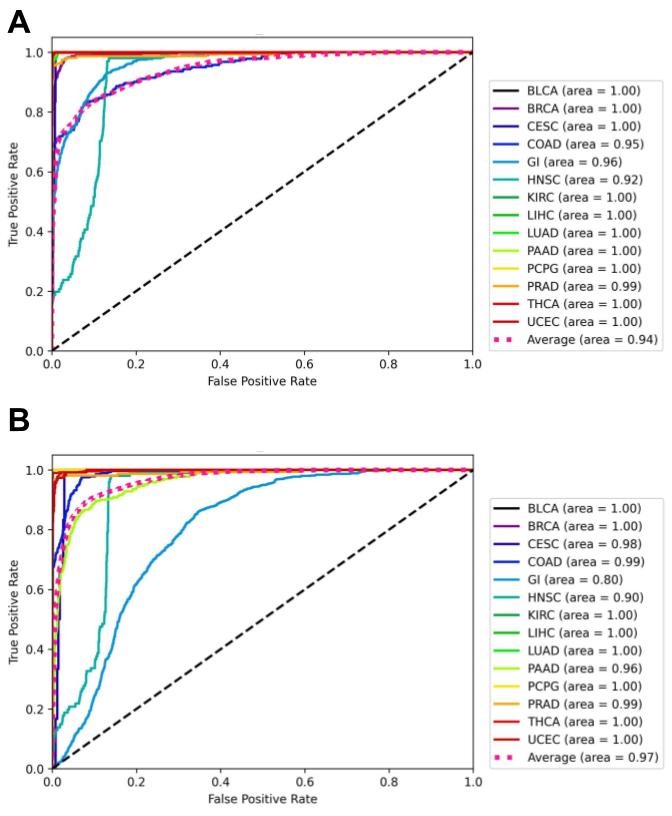


**[Figure S7] Comparison of AUROC between original and best-performing datasets.** The AUROC of each class and the micro-average of the AUROC for all classes after the classifiers were loaded with either the original datasets (**A** | *Unnormalized, No batch correction, Unscaled*) or the best-performing modified datasets (**B** | *QN, Batch correction, Scaled*). The training and independent test datasets were TCGA and GTEx, respectively. The batch effect correction algorithm used was Limma and all three types of batches (*Protocol batch effect, Disease batch effect, and Consortium batch effect*) were adjusted. These plots were taken from one of the models evaluated from the outer folds of cross-validation.


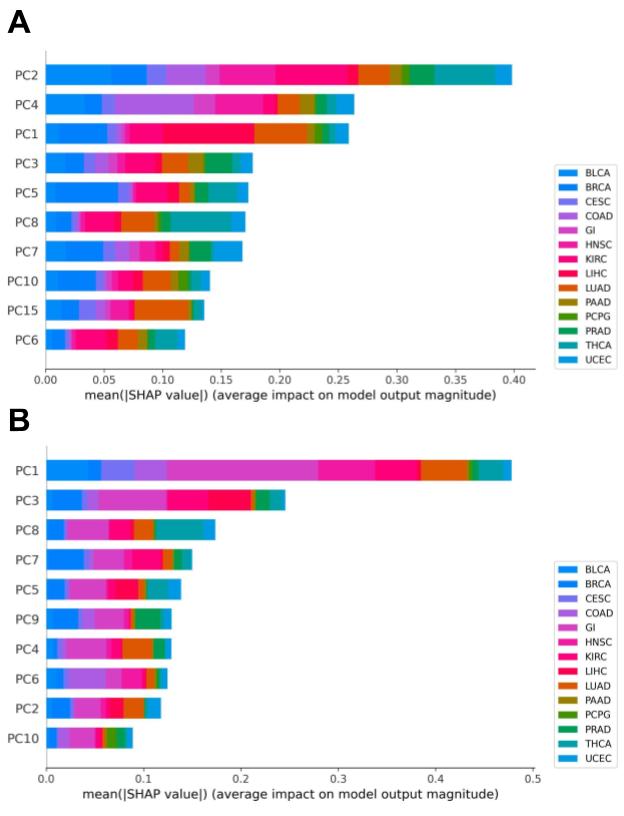


**[Figure S8] Comparison of SHAP global feature importance plots between original and best-performing datasets.** The SHAP values were calculated to determine the global importance of each PCA feature for the classifiers loaded with either the original datasets (**A** | *Unnormalized, No batch correction, Unscaled*) or the best-performing modified datasets (**B** | *QN, Batch correction, Scaled*). The training and independent test datasets were TCGA and GTEx, respectively. The batch effect correction algorithm used was Limma and all three types of batches (*Protocol batch effect, Disease batch effect, and Consortium batch effect*) were adjusted. The top ten PCA features are shown based on the mean absolute value for that feature over all the given samples. These plots were taken from one of the models evaluated from the outer folds of cross-validation.


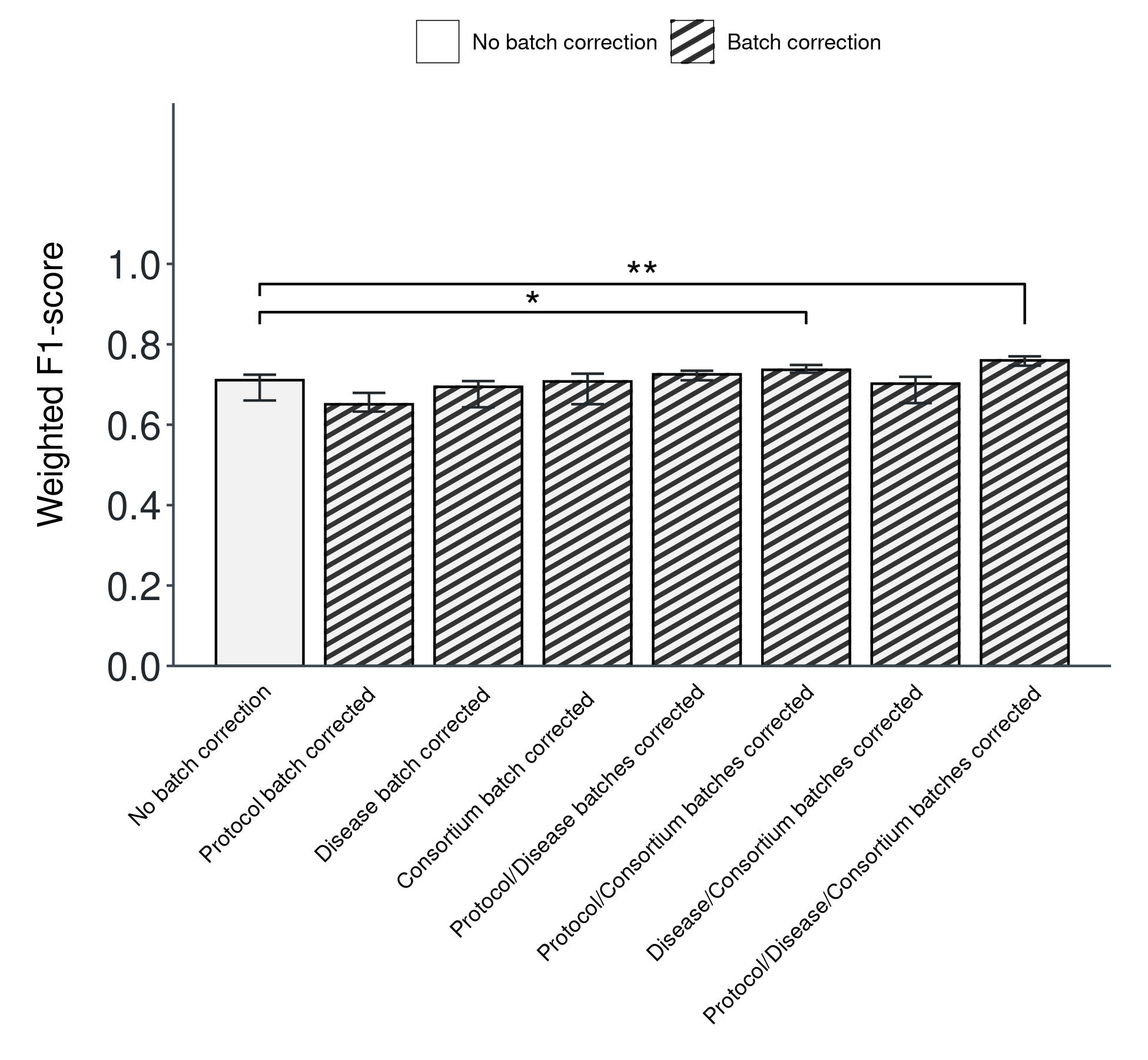


**[Figure S9] Batch effect adjustments are required for protocol and consortium batch effects to improve overall performance.** Weighted F1-scores as determined by SVM classifier loaded with the original dataset (left-most bar) versus the modified datasets after specific variations of batch effect corrections on the three types of batches (*Protocol batch effect, Disease batch effect, and Consortium batch effect*). All datasets were *Unnormalized* and *Unscaled*. The training and independent test datasets were TCGA and GTEx, respectively. The batch effect correction algorithm used was Limma. Bars indicate the median values of each group that consisted of five models evaluated from the outer folds of cross-validation. Error bars represent the 95% confidence interval. Statistical significance was determined with the Student's *t*-test. **p* < 0.05; ***p* < 0.01; ****p* < 0.001.


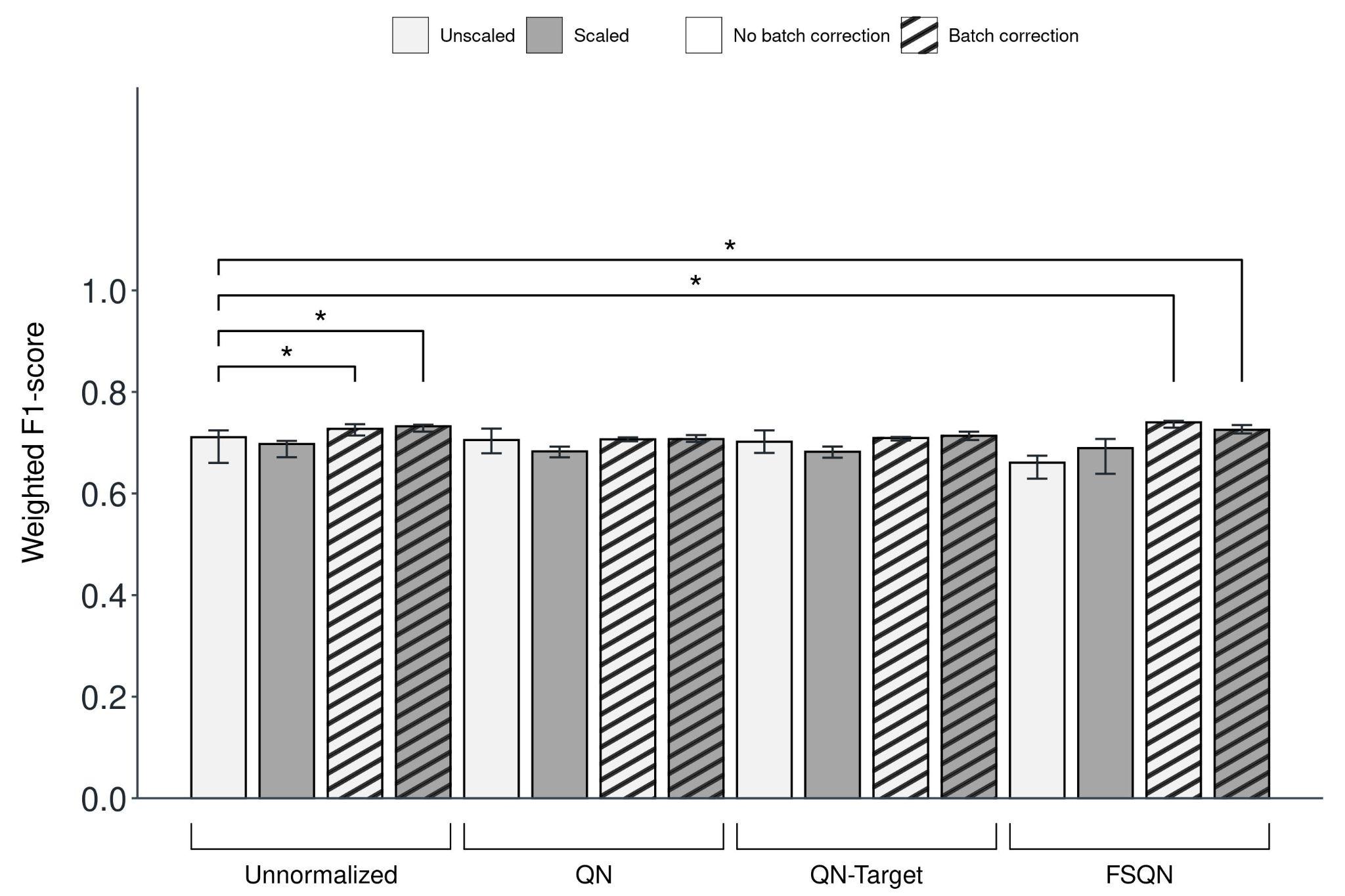


**[Figure S10] Limma's batch effect correction algorithm performed better than ComBat.**  Weighted F1-scores as determined by SVM classifier loaded with the original dataset (left-most bar) versus the modified datasets after combinations of normalization (*Unnormalized, QN [Quantile Normalization]*, *QN-Target [Quantile Normalization with Target], FSQN [Feature-Specific Quantile Normalization])*, batch effect correction (*No batch correction, Batch correction)* and scaling (*Unscaled, Scaled*) for default ComBat. The training and independent test datasets were TCGA and GTEx, respectively. All three types of batches (*Protocol batch effect, Disease batch effect, and Consortium batch effect*) were adjusted. Bars indicate the median values of each group that consisted of five models evaluated from the outer folds of cross-validation. Error bars represent the 95% confidence interval. Statistical significance was determined with the Student's *t*-test. **p* < 0.05; ***p* < 0.01; ****p* < 0.001.


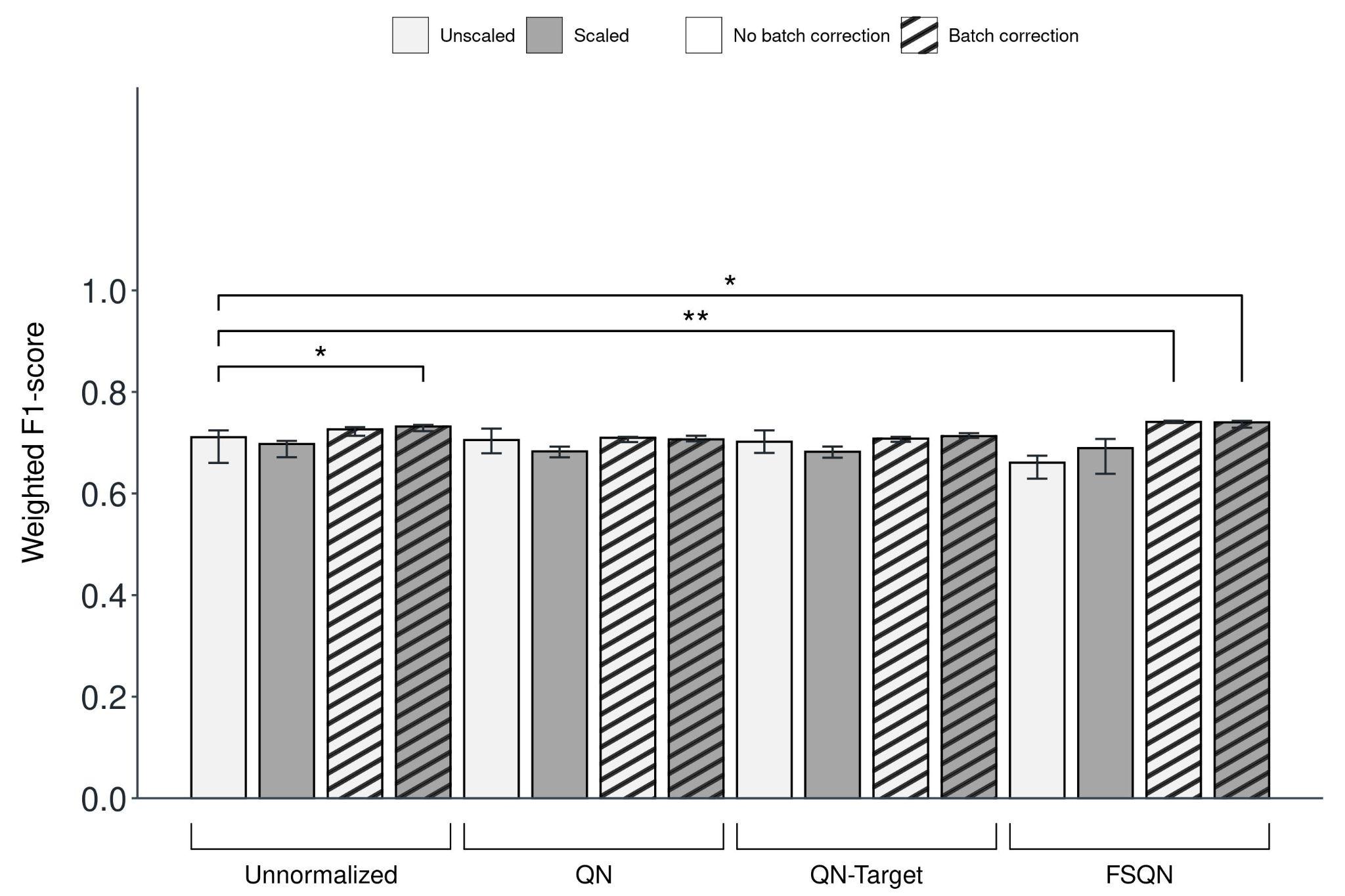


**[Figure S11] Limma's batch effect correction algorithm performed better than reference-batch ComBat.**  Weighted F1-scores as determined by SVM classifier loaded with the original dataset (left-most bar) versus the modified datasets after combinations of normalization (*Unnormalized, QN [Quantile Normalization]*, *QN-Target [Quantile Normalization with Target], FSQN [Feature-Specific Quantile Normalization])*, batch effect correction (*No batch correction, Batch correction)* and scaling (*Unscaled, Scaled*) for reference-batch ComBat. The training and independent test datasets were TCGA and GTEx, respectively. All three types of batches (*Protocol batch effect, Disease batch effect, and Consortium batch effect*) were adjusted. Bars indicate the median values of each group that consisted of five models evaluated from the outer folds of cross-validation. Error bars represent the 95% confidence interval. Statistical significance was determined with the Student's *t*-test. **p* < 0.05; ***p* < 0.01; ****p* < 0.001.
