# Supplementary material for: A comparison of RNA-Seq data preprocessing pipelines for transcriptomic predictions across independent studies
Source: BMC Bioinformatics. 2024 May 8;25:181. doi: 10.1186/s12859-024-05801-x (PMC11080237; doi:10.1186/s12859-024-05801-x)
Supplement: Supplementary file 9 — Additional file 9. [file 12859_2024_5801_MOESM9_ESM.docx]

| **Table S1. Summary of GEO datasets used in this study** | | | | | |
| --- | --- | --- | --- | --- | --- |
| **Dataset** | **Authors** | **Tissue Type** | ***n* Cancer samples** | ***n* Normal samples** | **Alignment tool** |
| GSE77509 | *Yang et al.* (76) | LIHC | 40 | 20 | TopHat (80) |
| GSE85465 | *Ooi et al.* (77) | GI | 4 | 4 | TopHat2 (81) |
| GSE85567 | *Nicodemus-Johnson et al.* (78) | LUAD | 0 | 85 | BWA (82) |
| GSE85606 | *Pidsley et al.* (79) | PRAD | 4 | 4 | TopHat2 (81) |

| **Table S2. Contribution of top genes to important PCA features based on SHAP analysis related to Figure S8** | | | | | | | |
| --- | --- | --- | --- | --- | --- | --- | --- |
| **Dataset** | **Rank** | **PCA feature** | **Top contributing genes** | | | | |
| Original | 1 | PC2 | *PI3* (1.98) | *KRT6A* (1.88) | *SFN* (1.73) | *GPX2* (1.69) | *KRT16* (1.66) |
|  | 2 | PC4 | *KRT5* (-1.76) | *KRT14* (-1.75) | *KRT6A* (-1.72) | *TSPAN8* (1.70) | *LGALS4* (1.66) |
|  | 3 | PC1 | *APOA2* (1.43) | *ALB* (1.42) | *SFRP2* (-1.37) | *APOC3* (1.36) | *ALDOB* (1.33) |
|  | 4 | PC3 | *IGKV4-1* (-1.18) | *IGHV5-51* (-1.14) | *IGKV3-20* (-1.14) | *IGHV3-23* (-1.14) | *IGHV1-18* (-1.13) |
|  | 5 | PC5 | *AZGP1* (-1.71) | *FOXA1* (-1.35) | *PAX8* (1.29) | *SPDEF* (-1.18) | *KCNJ16* (1.09) |
|  | 6 | PC8 | *SFTPB* (1.32) | *TG* (1.30) | *NKX2-1* (1.22) | *SFTA3* (1.09) | *RPL21P78* (0.99) |
|  | 7 | PC7 | *DES* (1.32) | *KLK3* (1.04) | *ACP3* (1.02) | *RPS4Y1* (1.02) | *PLA2G2A* (0.92) |
|  | 8 | PC10 | *CA12* (-0.81) | *TSPAN1* (-0.81) | *PDZK1IP1* (-0.74) | *CHGA* (0.73) | *CXCL14* (-0.70) |
|  | 9 | PC15 | *SFTPA2* (0.91) | *SFTPB* (0.87) | *SFTPA1* (0.83) | *PGC* (0.69) | *IGHA2* (-0.68) |
|  | 10 | PC6 | *SFTPB* (1.12) | *IGHA1* (1.03) | *C16orf89* (0.98) | *IGKC* (0.98) | *SLC34A2* (0.94) |
| Best-performing modified | 1 | PC1 | *KRT6A* (0.19) | *HPN* (-0.18) | *HJURP* (0.18) | *AURKB* (0.18) | *CENPA* (0.18) |
|  | 2 | PC3 | *NR1H4* (0.18) | *UGT2B7* (0.18) | *ARSL* (0.17) | *SLC39A5* (0.17) | *ACMSD* (0.17) |
|  | 3 | PC8 | *NKX2-1* (0.16) | *SFTA3* (0.16) | *NKX2-1-AS1* (0.15) | *HOXC10* (-0.14) | *EMX2* (-0.13) |
|  | 4 | PC7 | *SPDEF* (-0.16) | *MLPH* (-0.13) | *AZGP1* (-0.12) | *WDR72* (0.12) | *FOXA1* (0.12) |
|  | 5 | PC5 | *FOLR1* (-0.13) | *PAX8* (-0.13) | *AZGP1P1* (0.12) | *AZGP1* (0.12) | *PPP1R14C* (-0.12) |
|  | 6 | PC9 | *RPS4Y1* (0.15) | *PRAC1* (0.14) | *KLK3* (0.13) | *KLK2* (0.12) | *ESR1* (-0.12) |
|  | 7 | PC4 | *NOTCH2NL* (0.15) | *C21orf140* (0.14) | *SMIM11* (0.13) | *ENSG00000100101* (0.12) | *U2AF1* (-0.12) |
|  | 8 | PC6 | *KRT14* (-0.12) | *KRT5* (-0.11) | *GPR87* (-0.11) | *CLCA2* (-0.10) | *NHERF4* (0.10) |
|  | 9 | PC2 | *POSTN* (-0.12) | *REX1BD* (0.12) | *RBMS2P1* (-0.12) | *ARHGAP31* (-0.12) | *TAF10* (0.12) |
|  | 10 | PC10 | *MAB21L2* (0.09) | *TACSTD2* (-0.09) | *PROX1* (0.09) | *AQP3* (-0.09) | *FARS2-AS1* (-0.09) |
| *Inside the parentheses denotes the principal component loading value* | | | | | | | |
